# Identification and characterization of FGFR2^+^ hematopoietic stem cell-derived fibrocytes as precursors of cancer-associated fibroblasts induced by esophageal squamous cell carcinoma

**DOI:** 10.1186/s13046-022-02435-w

**Published:** 2022-08-08

**Authors:** Haibo Qiu, Xu Zhang, Jiali Qi, Jiangwen Zhang, Yin Tong, Lei Li, Li Fu, Yan-Ru Qin, Xinyuan Guan, Liyi Zhang

**Affiliations:** 1 Department of Gastric Surgery, State Key Laboratory of Oncology in South China, Sun Yat-Sen University Cancer Center, Collaborative Innovation Center for Cancer Medicine, Guangzhou, 510060 Guangdong People’s Republic of China; 2 Department of Thoracic Surgery, State Key Laboratory of Oncology in South China, Sun Yat-Sen University Cancer Center, Collaborative Innovation Center for Cancer Medicine, Guangzhou, 510060 Guangdong People’s Republic of China; 3 Department of Clinical Oncology, the University of Hong Kong, Hong Kong, 999077 People’s Republic of China; 4 School of Biological Sciences, the University of Hong Kong, Hong Kong, 999077 People’s Republic of China; 5 State Key Laboratory of Oncology in South China, Sun Yat-Sen University Cancer Center, Collaborative Innovation Center for Cancer Medicine, Guangzhou, 510060 Guangdong People’s Republic of China; 6 Shenzhen Key Laboratory of Translational Medicine of Tumor and Cancer Research Centre, Shenzhen University, Shenzhen, 518052 Guangdong People’s Republic of China; 7 Department of Clinical Oncology, the First Affiliated Hospital, Zhengzhou University, Zhengzhou, 450003 Henan People’s Republic of China

**Keywords:** CAF, FGFR2, CAF mobilization, CAF recruitment, CAF maturation, FGF2, CXCL12, CXCR4 axis, YAP-TEAD complex, Tumor microenvironment, ESCC

## Abstract

**Background:**

Cancer-associated fibroblast (CAF) is an ideal target for cancer treatment. Recent studies have focused on eliminating CAFs and their effects by targeting their markers or blocking individual CAF-secreted factors. However, these strategies have been limited by their specificity for targeting CAFs and effectiveness in blocking widespread influence of CAFs. To optimize CAF-targeted therapeutic strategies, we tried to explore the molecular mechanisms of CAF generation in this study.

**Methods:**

Using FGFR2 as a tracing marker, we identified a novel origin of CAFs in esophageal squamous cell carcinoma (ESCC). Furthermore, we successfully isolated CAF precursors from peripheral blood of ESCC patients and explored the mechanisms underlying their expansion, recruitment, and differentiation via RNA-sequencing and bioinformatics analysis. The mechanisms were further verified by using different models both in vitro and in vivo.

**Results:**

We found that FGFR2^+^ hematopoietic stem cell (HSC)-derived fibrocytes could be induced by ESCC cells, recruited into tumor xenografts, and differentiated into functional CAFs. They were mobilized by cancer-secreted FGF2 and recruited into tumor sites via the CXCL12/CXCR4 axis. Moreover, they differentiated into CAFs through the activation of YAP-TEAD complex, which is triggered by directly contracting with tumor cells. FGF2 and CXCR4 neutralizing antibodies could effectively block the mobilization and recruitment process of FGFR2^+^ CAFs. The YAP-TEAD complex-based mechanism hold promise for locally activation of genetically encoded therapeutic payloads at tumor sites.

**Conclusions:**

We identified a novel CAF origin and systematically studied the process of mobilization, recruitment, and maturation of CAFs in ESCC under the guidance of tumor cells. These findings give rise to new approaches that target CAFs before their incorporation into tumor stroma and use CAF-precursors as cellular vehicles to target tumor cells.

**Supplementary Information:**

The online version contains supplementary material available at 10.1186/s13046-022-02435-w.

## Background

It has become clear that cancer is not solely a disease governed by genetic and epigenetic alterations within tumor cells, but a disease directed by the body’s systemic responses to malignancy. Tumor cells play the leading role in cancer development and progression, while, other cells such as endothelial cells, immune cells, and fibroblasts are recruited into tumor beds and educated to facilitate the malignant progression [1]. These infiltrating cells have become attractive targets and tools for cancer treatment, because of their potential to destroy or encapsulate tumor cells in their local environment, as well as their genetic stability compared with frequently mutant tumor cells, which may bode well for a durable therapeutic response with very low probability of acquired resistance [2, 3]. At present, strategies targeting endothelial cells and immune cells have been applied to cancer therapy and achieved notable successes in some cancers [2, 4].

Recent attention has focused on cancer-associated fibroblasts (CAFs), the major cellular component of tumor microenvironment (TME). They have been reported to participate in tumor-stroma co-evolution and metastatic niche preparation by producing a variety of paracrine factors and creating proper extracellular matrix (ECM) structure for TME [5, 6]. Initially, most therapeutic studies concentrated on eliminating CAFs by targeting their markers, such as FAP and α-SMA. However, these strategies resulted in severe systemic toxicity, since the markers were not as specific for CAFs as assumed [7, 8]. Currently, researchers were enthusiastic about identifying specific inhibitors to block CAF-secreted factors. But the widespread influence of CAFs on TME has made these strategies difficult to succeed. For example, blocking CAF-derived MMPs, while showing promise in preclinical studies, was demonstrated to have nearly no beneficial response in Phase III clinical trials [9]. It means blocking individual products of CAFs is not enough. Nevertheless, targeting CAFs and their precursors before their incorporation into the tumor stroma may lead to a more encompassing downstream inhibition of CAF-related effects. Therefore, a deeper understanding of CAFs including their origins as well as the molecular mechanisms related to their recruitment and maturation is necessary.

In our previous study, we found that FGFR2 (FGFR2 mentioned in our study is FGFR2-IIIc) was a specific surface marker of CAFs in esophageal squamous cell carcinoma (ESCC) [10]. We have demonstrated that FGFR2^+^ CAFs actively interacted with ESCC cells through secreting multiple factors to support tumorigenesis and promote metastasis [11–14]. However, their precise origin and mechanisms about their recruitment and maturation are completely unknown. There are several theories regarding the origins of CAFs, and this topic is still under debate. Until now, several kinds of cells have been reported as possible precursors of CAFs, such as resident tissue fibroblasts, mesenchymal stem cells (MSCs), epithelial cells, and endothelial cells [5, 15]. Nevertheless, in esophageal cancer, an important clue has pointed that bone marrow (BM)-derived cells are a potential source of CAFs, as esophageal chronic inflammation promotes cancer development while chronic inflammation mediated BM-derived cell recruitment facilitates esophageal tissue repair [16]. Given the fact that FGFR2, the specific marker of CAFs in ESCC, is also a marker of some kind of progenitor cells in BM, it is reasonable to hypothesize that CAFs in ESCC may originate from BM-derived cells [17, 18]. In this study, we tested this hypothesis using a marker-based method, explored the molecular mechanisms of CAF generation mainly through high-throughput RNA-sequencing (RNA-seq) and bioinformatics analysis, and further verified the mechanisms by using different models both in vitro and in vivo.

## Material and Methods

### Cell lines

Japanese ESCC cell lines (KYSE30 and KYSE140) were obtained from DSMZ, the German Resource Centre for Biological Material. Chinese ESCC cell lines (EC9706 and HKESC1) and normal esophageal epithelial cell lines (NE1) were kindly provided by Professor George Sai Wah Tsao (Department of Anatomy, the University of Hong Kong, Hong Kong, China). All cell lines were authenticated by short-tandem repeat analysis. ESCC cells were cultured in DMEM (Life Technologies, Gaithersburg, MD, USA) supplemented with 10% FBS (Thermo Fisher Scientific, San Jose, CA, USA) and penicillin–streptomycin (Life Technologies, Gaithersburg, MD, USA) at 37ºC in 5% CO_2_. Normal epithelial cells were cultured in a 1:1 mixture of defined keratinocyte serum-free medium (Invitrogen, Mount Waverley, VIC, Australia) and EpiLife (Cascade Biologics, Portland, Oregon, USA) at 37ºC in 5% CO_2_.

### Primary cells

BM cells for immunofluorescence (IF) staining or flow cytometry (FCM) analysis were collected by flushing femurs of euthanized nude mice with Hank's balanced salt solution (Thermo Fisher Scientific, San Jose, CA, USA). Leukocyte samples for FCM analysis were prepared by depleting erythrocytes. Briefly, blood was mixed with 20 volume of 1 × FACS lysing solution (BD Biosciences, San Jose, CA, USA), incubated on ice for 10 min to lyse erythrocytes, and washed twice at 4 °C with PBS.

Fibroblasts (normal fibroblasts [NFs]/CAFs from ESCC patients and induced-CAFs [iCAFs] from tumor-bearing mice with intravenous [i.v.] injections of FGFR2^+^ fibrocytes) were isolated from normal esophageal epithelium, ESCC tissue specimens or tumor xenografts, respectively. In short, freshly collected tissues were cut into as small pieces as possible in sterile PBS, followed by collagenase digestion (Thermo Fisher Scientific, San Jose, CA, USA). The suspension was filtered through 20 µm stainless steel wire mesh to collect a single cell suspension. The filtrate was centrifuged and washed before being finally plated on 6 cm tissue culture dishes in 5 mL DMEM medium supplemented with 10% FBS. After culturing for 30 min at 37ºC, unadherent cells (mainly tumor cells) were removed to obtain pure fibroblasts. The adherent fibroblasts were subcultured for further study.

Fibrocytes (human and mouse) were purified from peripheral blood. Briefly, peripheral blood mononuclear cells (PBMCs) were isolated by centrifugation over Ficoll (GE Healthcare, Milwaukee, WI, USA) following the protocol of manufacturer and cultured on Fibronectin pre-coated tissue culture plates (BD Biosciences, San Jose, CA, USA) in DMEM supplemented with 10% FBS. After 2 days, the nonadherent cells (largely lymphocytes) were aspirated off, and the remaining adherent cells cultivated for 14 days. Over time, the contaminating monocytes died off, and fibrocytes appeared as spindle shaped cells (Fig. S1A). The crude fibrocytes were lifted by incubation in trypsin–EDTA 0.05% (Thermo Fisher Scientific, San Jose, CA, USA) and purified by immunomagnetic selection using anti-Col I microbeads (Miltenyi Biotec, Bergisch Gladbach, Germany). The resultant cultured, enriched fibrocyte populations were > 94% pure based on FGFR2 and CD34/CD45/Col I staining (Fig. S1B). Typically, between 2 and 8 × 10^4^ fibrocytes were isolated per milliliter of peripheral blood.

### Mice

Athymic BALB/C nude mice were housed under standard conditions and cared for according to the institutional guidelines for animal care. All animal procedures were performed under Hong Kong Animals (Control of Experiments) Ordinance license (Chapter 340) and with approval from the University of Hong Kong Animal Welfare Committee.

To determine the origin of FGFR2^+^ CAFs, ESCC tumor-bearing models were constructed using nude mice. 1 × 10^6^ ESCC cells (KYSE30, EC9706, KYSE140, or HKESC1) were subcutaneous (s.c.) injected into the left hind legs of 4-week-old nude mice, respectively. Five mice per group were used to monitor FGFR2^+^ cells in peripheral blood. Three mice per group were used to identify the cell type of FGFR2^+^ cells in peripheral blood by two-color FCM. Besides, 24 mice per group were used for BM analysis. Day of inoculation of tumors in mice was considered to be day 0 (week 0). Mice were followed for 7 to 8 weeks after injection of tumor cells. Xenograft tumors were removed at week 4 after injection. The surgical procedure was mimicked by making a 1 cm long skin incision in control mice without observable tumors (mice injected with PBS or KYSE140).

The mice used to perform in vivo chemotaxis assays were s.c. injected with 1 × 10^6^ ESCC cells (KYSE30 or EC9706) one week before i.v. injection of FGFR2^+^ fibrocytes. Three mice will be evaluated for each condition.

To examine the effect of iCAFs on tumorigenicity, 1 × 10^6^ ESCC cells (KYSE30 or EC9706) were s.c. injected into nude mice one week before i.v. injection of FGFR2^+^ fibrocytes. Tumor formation in nude mice was monitored over a 15-day period. Tumor size was measured every 3 days. The tumor volume was calculated by the formula V = 0.5 × L × W^2^. Four mice will be evaluated for each condition.

In FGF2 or CXCR4 inhibition assay, tumor-bearing mice were also established by s.c. injected with 1 × 10^6^ ESCC cells (KYSE30 or EC9706). For FGF2 inhibition, mice were i.v. injected with 4 µg/kg FGF2 neutralizing antibody every day. Four mice per group were used. Tumor size was monitored every 4 days over a 20-day period. Each tumor xenograft was dissected, weighed, fixed in 4% paraformaldehyde, embedded in paraffin, and processed for hematoxylin/eosin staining and immunohistochemistry (IHC) analysis. For CXCR4 inhibition, mice were treated with 1, 5, or 10 µg CXCR4 neutralizing antibody every day over a 7-day period. Four mice per group were used. Each tumor xenograft was collected and processed for IHC staining. The mice treated with PBS or isotype-matched immunoglobulins were served as controls. Neutralizing antibodies used in these studies were listed in Table S1.

### Human blood samples and tissue specimens

Blood samples of healthy controls and ESCC patients were kindly provided by Sun Yat-Sen University Cancer Center (Guangzhou, Guangdong, China). All blood samples from ESCC patients were collected before surgical resection. Primary ESCC tumors and adjacent nontumorous tissues from the proximal resection margins were collected immediately after surgical resection at Sun Yat-Sen University Cancer Center. Pairs of ESCC RNA samples and their corresponding RNA samples from nontumorous tissue were kindly provided by the First Affiliated Hospital, ZhengZhou University (Zhengzhou, Henan, China). No patients recruited in this study have received any preoperative treatment. Informed consent was obtained from all patients before the collection of blood samples and tissue specimens, and the study was approved by the Committees for Ethical Review of Research involving Human Subjects at Sun Yat-Sen University and Zhengzhou University.

### FCM analysis

FCM analysis was conducted using antibodies with fluorescein conjugates. Cells were incubated in PBS containing 2% FBS with either FITC-conjugated primary antibody and/or PE-conjugated primary antibody. Isotype-matched mouse and/or rabbit immunoglobulin were served as controls to gate positive cells. Samples were analyzed on BD LSR Fortessa Analyzer (BD Biosciences, San Jose, CA, USA), and data were analyzed using FlowJo software (Tree Star, San Carlos, CA, USA). Antibodies used in this study were listed in Table S2.

### IF

Before processing, fibrocytes and fibroblasts (NFs, CAFs and iCAFs) were seeded on cover slips at 25%-50% confluency, and BM cells were centrifuged onto glass slides using a Cytospin centrifuge (800 g for 5 min). The slides were fixed in 4% paraformaldehyde for 10 min and permeabilized in 0.05% Triton X-100 for 1–5 min. After washing in PBS, primary antibodies with or without fluorescein conjugates were added and the slides were incubated at 4 °C overnight in a dark humidified chamber. On the second day, primary antibodies were washed away. The slides treated with primary antibodies without fluorescein conjugates were incubated with FITC- or PE-conjugated secondary antibodies for 30 min. The nucleus was stained by DAPI. All slides were examined at a magnification of 40 × in a fluorescence microscope (Carl Zeiss LSM 710). Primary antibodies used in this study were listed in Table S2.

### IHC

Standard streptavidin–biotin-peroidase complex method was used for IHC staining. Briefly, xenograft and organ sections were deparaffinized in xylene and rehydrated in graded alcohols and distilled water. Slides were heated for antigen retrieval in 1 × retrieval buffer (Dako, Palo Alto, CA, USA). The EnVision Plus System (Dako, Palo Alto, CA, USA) was used for IHC according to the manufacturer’s instructions. Sections were incubated with anti-FGFR2 or anti-Luciferase antibody overnight at 4 °C. Stained slides were imaged on an Aperio Scanscope CS imager (Leica Biosystems, Newcastle, UK). The immunostaining area of FGFR2 or Luciferase was quantified by Image-Pro Plus 6.0 software (Media Cybernetics, Silver Spring, MD, USA). Primary antibodies used in this study were listed in Table S2.

### Generation of target gene-overexpression/knockdown cells

GFP-expression plasmid (pLenti6-CMV-MCS-GFP-SV-puro, Invitroge), Luciferase-expression plasmid (pLV-EF1a-Firefly Luciferase-IRES-Bsd, GeneCopoeia) or pLV-EF1a empty vector with blasticidin selectable marker (GeneCopoeia, Rockville, MD, USA) were packaged using Lentiviral Packaging mix (GeneCopoeia, Rockville, MD, USA) and used to infect ESCC cell lines or FGFR2^+^ fibrocytes to establish ESCC cells constitutively expressing GFP and fibrocytes constitutively expressing Luciferase or resistant to blasticidin. Stable clones were selected using puromycin or blasticidin and subjected to in vitro experiments.

To generate CXCR4, YAP, or TEADs knockdown cells, oligonucleotides directly against these genes were cloned into pLKO.1, respectively (short hairpin RNAs [shRNAs] of TEADs were designed in a region identical in TEAD1, 2, 3 and 4). The sequences of the oligonucleotides were summarized in Table S3. Plasmids were propagated in and purified from Stbl3 competent cells (Invitrogen, Mount Waverley, VIC, Australia). shRNA expression plasmid and lentiviral packaging plasmids were co-transfected into HEK293-T cells for virus production using Lipofectamine 2000 (Invitrogen, Mount Waverley, VIC, Australia). Fibrocytes or CAFs were infected with lentiviral media in the presence of 10 µg/mL polybrene (Sigma-Aldrich, St Louis, MO, USA) overnight at 37ºC in 5% CO_2_. The cells with stable target gene silencing were selected using puromycin and then collected for RNA extraction and/or subjected to in vitro experiments. The cells infected with non-targeting shRNA were served as controls.

### In vivo chemotaxis assay

To evaluate the chemotaxis capability of FGFR2^+^-circulating fibrocytes, Luciferase-expressing fibrocytes (2 × 10^5^ per mice) were i.v. injected into ESCC tumor-bearing mice (tumors were formed by GFP-expressing KYSE30 or EC9706). After 24 h, fibrocyte infiltration (luminescent signals) and tumor burden (fluorescence signals) were assessed via in vivo fluorescence and bioluminescence measurement using IVIS Imaging System (Perkin Elmer, Heidelberg, Germany). Before acquisition of bioluminescence images, D-luciferin (50 mg/kg) was intraperitoneally injected to tumor-bearing mice. All of the bioluminescence images were acquired using the same parameters: Excitation filter (Block), Emission filter (Open), Exposure time = 5 min, Binning = Medium, F/Stop = 1. Sequentially, fluorescence images were acquired using the same parameters besides the Excitation filter (465 nm), Emission filter (520 nm), Exposure time = 1 s. Image analysis was performed with Living Image 4.4. A region of interest (ROI) was manually selected over signal intensity. The area of the ROI was kept constant. The in vivo chemotactic index was calculated as relative radiance (Radiance of Luminescent in ROI/Radiance of Fluorescence in ROI). The nontumor-bearing mice i.v. injected with fibrocytes were used as control. The tumor-bearing mice i.v. injected with PBS were used to discard non-specific background signals in bioluminescence detection. Three nude mice will be evaluated for each condition.

### In vitro chemotaxis assay

Chemotaxis of fibrocytes was assayed in a transwell system (Corning, Cambridge, MA, USA) using 8 μm polycarbonate membranes precoated with Matrigel (BD Biosciences, San Jose, CA, USA). 800 μL serum-free DMEM with ESCC cells (KYSE30 or EC9706, 1 × 10^5^ per well) or CXCL12 recombinant proteins (0.01, 0.1 or 1 ng/mL) was added to the lower chamber as chemotactic stimulus. Fibrocytes (5 × 10^3^ per well) suspended in 500 μL serum-free DMEM alone or with CXCR4 neutralizing antibody (0.2, 1, or 5 μg/mL) were loaded into the upper chamber and incubated for 24 h at 37 °C, 5% CO_2_. Fibrocytes migration toward DMEM alone or DMEM with isotype-matched immunoglobulin was used as the negative control. Invasive fibrocytes were fixed, stained and counted under a microscope. The chemotactic index was calculated as the number of cells invaded per 40 × field. Three independent experiments were done with triplicates each time. Recombinant proteins and neutralizing antibodies used in this study were listed in Table S1.

### In vivo differentiation assay

To induce FGFR2^+^ fibrocytes differentiation in vivo, 2 × 10^5^ FGFR2^+^-blasticidin resistant-fibrocytes were i.v. injected into ESCC tumor-bearing mice. One week later, exogenous cells were isolated from freshly collected xenografts. The isolation process was similar to that of fibroblast isolation except that the isolated cells were treated with blasticidin (Sigma-Aldrich, St Louis, MO, USA) for selection of exogenous cells. These cells were collected for RNA extraction after 4-day selection, characterization was performed by semi-quantitative PCR (qPCR), which detects the expression of fibrocyte, fibroblast and CAF marker genes. FGFR2^+^ fibrocytes cultured in normal culture medium and FGFR2^+^-blasticidin resistant-fibrocytes cultured in medium with blasticidin were served as controls. Three independent experiments were done.

### In vitro differentiation assay

To induce FGFR2^+^ fibrocytes differentiation in vitro, we used three distinct co-culture systems. For system I, FGFR2^+^ fibrocytes were seeded into 6-well plate at a density of 2 × 10^4^ per well and cultured in conditioned medium from KYSE30. After 48 h, the cells were collected for RNA extraction. Fibrocytes treated with normal culture medium or conditioned medium from NE1 were used as control. In system II, FGFR2^+^ fibrocytes and KYSE30 cells were co-cultured for 48 h in the same well, but physical interaction was prevented by growing the KYSE30 cells in Millicell hanging inserts (Millipore, Bedford, MA, USA), which are inserted into the wells of 6-well plates and sit 1-2 mm above the layer of fibrocytes. The bottom of the insert is formed by a cellulose membrane containing 0.45 µm pores, which allow for diffusion of soluble effectors. Fibrocytes cultured in normal culture medium or co-culture with NE1 were served as control. For system III, directly co-cultured of FGFR2^+^-blasticidin resistant-fibrocytes and KYSE30 cells were established in the same well at ratio of 1:5 (fibrocyte/KYSE30). After 48 h, the culture medium was complete removed, whereas, the cells were continually cultured in medium with blasticidin for 48 h before RNA extraction. Over time, all KYSE30 cells died off, and fibrocytes were left. Fibrocytes cultured in normal culture medium, blasticidin resistant-fibrocytes cultured in medium with blasticidin or co-culture with NE1 were used as control. For preparation of conditioned medium or feeder cells in co-culture systems, the KYSE30 and NE1 cells were mitotically inactivated in a medium containing 10 μg/mL mitomycin C for 24 h. The cell identities in differentiation assays were accessed as described previously. All Experiments were done in triplicate.

### qPCR

Total RNA was extracted from cells and frozen tissues by the TRIzol reagent (Invitrogen, Mount Waverley, VIC, Australia). Reverse transcription of total RNA was done using a PrimeScript RT reagent Kit (Clontech, Palo Alto, CA, USA), and complementary DNA (cDNA) was subjected to qPCR using SYBR Green master mix (Roche, Lilleroe, Denmark) with housekeeping gene β-actin as an internal control. qPCR was performed on HT7900 system (Applied Biosystems, Foster City, CA, USA). Primers used were listed in Table S4.

### XTT cell proliferation assay

To examine the effect of conditioned medium from iCAFs on growth of ESCC cells, KYSE30 or EC9706 cells were seeded into 96-well plate at a density of 1.0 × 10^3^ per well and cultured in conditioned medium from iCAFs. Conditioned medium from fibrocytes or normal culture medium was used as control. The cell growth rate was determined by XTT Cell Proliferation Kit II (Roche, Lilleroe, Denmark) according to the manufacturer’s instruction. Three independent experiments were done with triplicates each time.

### Transwell invasion assay

To examine the effect of conditioned medium from iCAFs on invasion of ESCC cells, KYSE30 or EC9706 cells (5 × 10^4^) were suspended in 0.5 ml serum-free conditioned medium from iCAFs and loaded on the upper invasion chamber coated with Matrigel (Corning, Cambridge, MA, USA). The lower chamber was filled with normal culture medium supplemented with 10% FBS.

After 48 h, invasive cells were fixed, stained and counted under a microscope. Results are expressed as the number of cells invaded per 40 × field. Three independent experiments were done with triplicates each time.

### Enzyme-linked immunosorbent assay (ELISA)

FGF2 concentrations in serum and CXCL12 concentrations in plasma or tissue homogenates were measured by FGF2/CXCL12 ELISA Kit (Cat# KHG0021, Life Technologies, Gaithersburg, MD, USA; Cat# DSA00, R&D Systems, Minneapolis, MN, USA) according to the manufacturer’s instruction, respectively. All Experiments were done in triplicate.

### RNA extraction and Illumina messenger RNA library preparation

Total RNA of fibrocytes and CAFs was isolated using the mirVana miRNA Isolation Kit (Applied Biosystems, Foster City, CA, USA). Extracted total RNA were treated with the DNA-free Kit (Ambion, Austin, TX, USA). Large 18S and 28S ribosomal RNAs (rRNAs) were removed from total RNA with the RiboMinus Transcriptome Isolation Kit (Invitrogen, Mount Waverley, VIC, Australia). The rRNA-depleted RNA was precipitated with Pellet Paint (Novagen Inc, Madison, WI, USA) and checked on the Agilent 2100 Bioanalyzer (Agilent Technologies, Palo Alto, CA, USA). The rRNA depleted RNA was fragmented by incubation for 5 min at 94 °C in 5 × Array Fragmentation Buffer (Ambion, Austin, TX, USA). The reaction was stopped by chilling the tube on ice and precipitated with Pellet Paint. cDNA was synthesized with the SuperScript Double Stranded cDNA Synthesis Kit (Invitrogen, Mount Waverley, VIC, Australia) using random hexamers. The reaction was purified on a QiaQuick PCR column (Qiagen, Hilden, Germany). Double-strand cDNA fragments were repaired with DNA Terminator End Repair Kit (Lucigen, Middleton, WI, USA) incubated for 30 min at 30 °C and purified on a QiaQuick PCR column. The Klenow 3’ to 5’ exo-polymerase (NEB, Ipswich, MA, USA) was used to add a single “A” base to the 3’ end of blunt phosphorylated DNA fragments by incubation for 30 min at 30 °C. Following purification, an Illumina PE Adapter (Illumina, San Diego, CA, USA) was ligated to the end of DNA fragments with the Quick Ligation Kit by incubation for 15 min at room temperature. The reaction was purified on a QiaQuick PCR column. 300 to 320-base pair fragments were excised from a 2% low-melting agarose gel. Fragments were enriched by 10 cycles using AccuPrime Pfx DNA Polymerase (Invitrogen, Mount Waverley, VIC, Australia). PCR product was run on Novex 8% TBE polyacrylamide gel (Invitrogen, Mount Waverley, VIC, Australia) and stained with SYBR Gold (Invitrogen, Mount Waverley, VIC, Australia). The gel slice containing the 300 to 320-base pair fragment was excised and the DNA purified using the QiaQuick Gel Extraction Kit (Qiagen, Hilden, Germany). The concentration of gel-purified DNA fragments was measured using an ND-1000 UV/Vis spectrophotometer (NanoDrop Technologies, Wilmington, DE, USA).

### RNA-seq

Cluster generation and sequencing were performed using the Standard Cluster Generation Kit v4 and TruSeq SBS Kit v3 on the Illumina Cluster Station and GAIIx following the manufacturer’s instructions (Illumina, San Diego, CA, USA). cDNA libraries from 2 paired fibrocytes and their corresponding CAFs (F1/C1 and F2/C2) were sequenced with 38-base single reads. Raw data from the GAIIx were analyzed with the Illumina Real Time Analysis (RTA) v1.6. A PhiX 174 control lane was included in each Solexa run for matrix, phasing, and error rate estimations as recommended by the manufacturer. The error rate of the PhiX control was < 0.1% for all the sequencing runs. Sequencing reads with polymer tracks longer than 30 base pairs or with primer sequences longer than 36 base pairs were filtered. Ribosomal RNA sequences were also removed by aligning to 28S (NCBI RefSeq accession number NR_003287.2), 18S (NCBI RefSeq accession number NR_003286.2), human ribosomal DNA complete repeating unit (HSU13369), and mitochondrial ribosomes (Ensembl transcript ID ENST00000387347 and ENST00000389680) with at least 95% similarity. The clean and high-quality reads were then mapped against human genome assembly (NCBI Build 37.1) using CLC Genomics Workbench (CLC Bio, Aarhus, Denmark). The 9 alternate assembly loci were excluded from the reference. Only sequencing reads with at most 2 mismatches per loci and a maximum of 10 mappable locations were retained for calculation of gene expression. All genes mapped by at least 10 reads in any of the 4 samples were considered. The expression level of each gene was measured by the number of reads per gene per kilobase exon per million mapped reads (RPKM) by normalizing the number of exon reads to the length of exons within that gene and per million mapped reads. The differential expression analysis was performed using Baggerley’s test. The genes significantly differentially expressed were extracted with a Benjamini–Hochberg corrected *P*-value < 0.005 for downstream GO and KEGG pathway analysis.

### Gene set enrichment analysis (GSEA)

RNA-seq data was processed and analyzed using the GSEA software, developed by the Broad Institute of MIT and Harvard (USA) and available at www.broadinstitute.org, following the program guidelines. The specific settings applied in all analyses are: Number of Permutations (1000), Permutation Type (Gene set), Enrichment statistic (Weighted), Metric for ranking genes (t Test). The rest of the fields were left as defaulted. Values in the tables represent the Normalized Enrichment Score and Nominal *P*-value of each gene set. The list of the specific gene sets analyzed and their sources are available in Table S5.

### Motif enrichment analysis

Putative promoter and enhancer regions of CAF-specific extracellular factor encoded genes were identified by FANTOM5 CAGE database, VISTA Enhancer Browser and ENCODE Encyclopedia. Motif enrichment in these regulatory regions was analyzed using HOMER version 4.9, a suite of tools for regulatory element analysis in genomics applications. Sequences of the two region sets (promoters and enhancers of CAF-specific extracellular factor encoded genes) were compared to background sequences, matched for GC content and auto-normalized to remove bias from lower-order oligo sequences. After masking repeats, motif enrichment was calculated using the cumulative binomial distribution by considering the total number of target and background sequence regions containing at least one instance of the motif. The top scoring motifs resulting from each search were combined, remapped and ranked according to enrichment in our promoter and enhancer region set after filtering redundant motifs. Their best-matching transcription factors were found by alignment. The predicted transcriptional regulatory networks were than constructed by Cytoscape (Table S6).

### Western blot

Denatured protein samples were loaded into the wells of 10% SDS-PAGE gel along with molecular weight marker. After electrophoresis, proteins were transferred to PVDF membranes with a constant voltage of 100 V for 2 h. The PVDF membranes were blocked with 5% non-fat milk for 1 h and incubated with primary antibody at 4℃ overnight. After washing in TBS-T, the PVDF membranes were incubated for 1 h with corresponding secondary antibody. Immune detection was performed using ECL Western Blotting Detection Kit and Hyperfilm. Primary antibodies used in this study were listed in Table S2.

### Statistical analysis

All statistical analyses were performed using SPSS statistical package for Windows, version 13.0. Data are represented as the mean ± SD from at least three independent experiments or 3 mice. The significance of the difference between groups was evaluated with the Student's t-test or χ2 test. *P*-value < 0.05 was considered significant. In experiments where there were multiple groups or variables, these were compared to the control for each sample using a 2-sample t-test. Due to the amount of data presented in this manuscript, we have highlighted the statistical significance for the most relevant comparisons.

## Results

### ESCC tumor implantation mobilizes FGFR2^+^ BM cells into peripheral blood

**Fig. 1:**
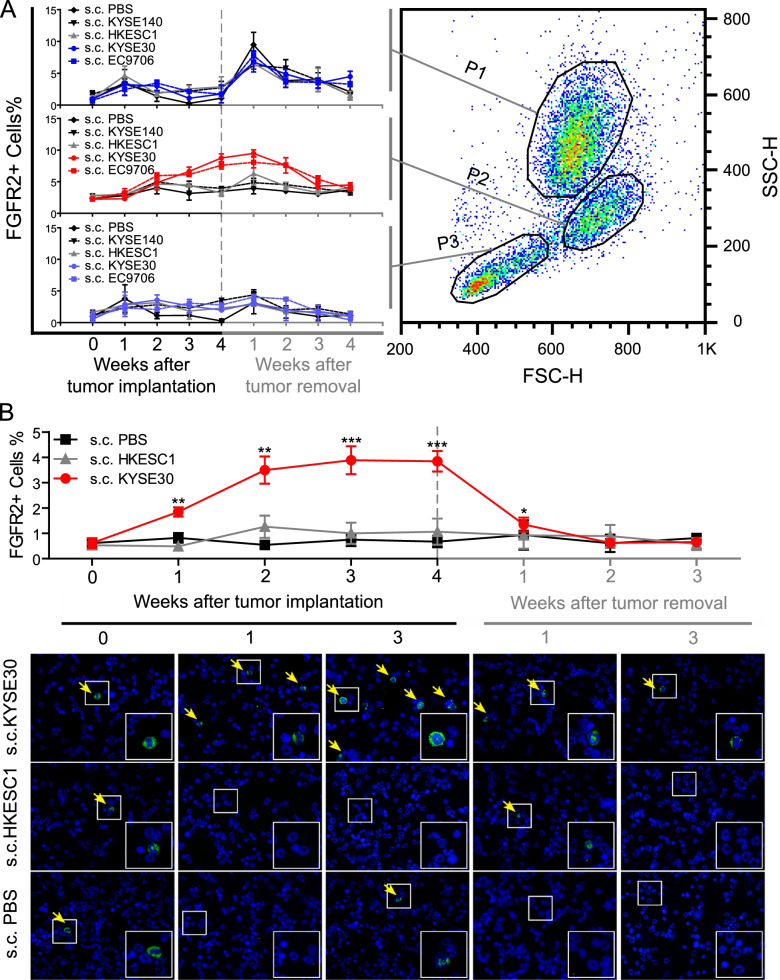
ESCC tumor implantation mobilizes FGFR2^+^ BM cells into peripheral blood. **A.** FGFR2^+^ cells in peripheral blood were monitored by FCM at a series of indicated time points after s.c. injection of ESCC cells or tumor resection. Based on size (FSC-H) and granularity (SSC-H), leukocytes were divided into P1, P2 and P3 (dot plot on the right). The percentage of FGFR2^+^ cells in each faction were measured and summarized in left panel. The data are represented as mean ± SD on 5 independent samples. **B.** FGFR2^+^ cells in BM were evaluated by FCM (upper panel) at a series of indicated time points after ESCC tumor implantation or resection. The data are represented as mean ± SD on 3 independent samples. *, *P* < 0.05; **, *P* < 0.01; ***, *P* < 0.001. Representative images of IF staining with FGFR2 antibody (green) in the same BM samples are shown in lower panel. Nuclei were counterstained with DAPI (blue). FGFR2^+^ cells are pointed by yellow arrows. Selected regions are magnified. (original magnification, 400 ×). Abbreviations: ESCC, esophageal squamous cell carcinoma; FCM, flow cytometry; BM, bone marrow; s.c., subcutaneous; SD, standard deviation; IF, immunofluorescence

If FGFR2^+^ CAFs are originated from BM, before arriving in tumor beds, they or their precursors should be mobilized in BM and thereafter introduced into peripheral circulation in response to ESCC stimuli. To examine this possibility, we constructed xenograft models utilizing two types of human ESCC cell lines. The first type, termed here “active cell lines”, including KYSE30 and EC9706, are the cell lines which yield vigorously growing tumor xenografts. These xenografts contain abundant FGFR2^+^ CAFs, indicating that they can recruit murine FGFR2^+^ CAFs. The second type, termed here “indolent cell lines”, including KYSE140, the cell line never able to form observable tumors, and HKESC1, the cell line with tumorigenicity but without the capacity to recruit FGFR2^+^ CAFs. FGFR2^+^ circulating cells in the nude mice with either active or indolent cells s.c. injection were then monitored by FCM. Based on physical gating, leukocyte pool can be divided into three main populations: granulocyte (P1), monocyte (P2) and lymphocyte (P3) (Fig. 1A). Before s.c. injection of tumor cells, FGFR2^+^ cells accounted for about 1.08 ± 0.50%, 2.50 ± 0.32% and 1.06 ± 0.58% in P1, P2, and P3, respectively. After tumor implantation, we found that all of the mice showed a slightly increase in P1 and P3-gated FGFR2^+^ cells during the first week, which was followed by a rapid decrease and then stayed constant at basal level over the next 3 weeks. However, a dramatic difference was observed in P2-gated FGFR2^+^ cells between active cell-treated mice and indolent cell/PBS-treated mice. FGFR2^+^ cells in P2 were gradually increased to 8.75 ± 1.05% and 7.60 ± 1.13% following KYSE30 and EC9706 injection, respectively. In contrast, after KYSE140, HKESC1 or PBS injection, P2-gated FGFR2^+^ cells remained stable at basal level (Fig. 1A). These findings suggested that FGFR2^+^ cells in P2 could be mobilized by active cells, but not by indolent cells. Besides, FGFR2^+^ cells in P1 and P3 were only associated with inflammatory response.

To confirm these findings, we removed the tumors from the same group of mice. As expected, P2-gated FGFR2^+^ cells were gradually decreased to basal level after active tumor removal, demonstrating that FGFR2^+^ cells in P2 were specifically mobilized by active tumors. Whereas, rapid increase of P1-gated FGFR2^+^ cells in the first week and rapid decrease in the second week after surgical resection confirmed that FGFR2^+^ cells in P1 were correlated with acute inflammation response (Fig. 1A).

Using the same animal model, we examined the impact of tumor implantation and resection on FGFR2^+^ cells in BM by FCM and IF. Consistent with the studies of FGFR2^+^ circulating cells, we found that injecting HKESC1 cells and PBS did not affect the frequency of FGFR2^+^ cells in BM. But implantation with KYSE30 could significantly increase FGFR2^+^ BM cells, which was rapidly decreased to basal level after tumor removal, indicating that FGFR2^+^ cells in BM could be mobilized by active tumors (Fig. 1B).

### FGFR2^+^ circulating cells are hematopoietic stem cell (HSC)-derived fibrocytes

**Fig. 2:**
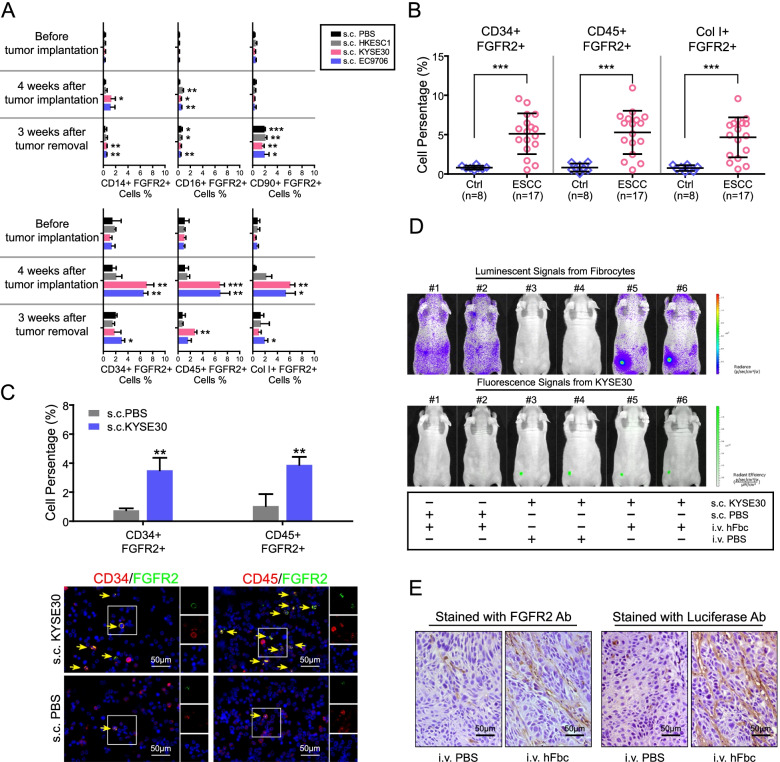
FGFR2^+^ CAFs are HSC-derived fibrocytes. **A.** Cell-type identification of FGFR2^+^ cells in peripheral blood from ESCC tumor-bearing mice. Leukocytes obtained at indicated time points after tumor implantation or resection were double-stained for FGFR2 in combination with indicated cell surface markers and analyzed by FCM. The data are represented as mean ± SD on 3 independent samples. *, *P* < 0.05; **, *P* < 0.01; ***, *P* < 0.001. **B.** Quantification of circulating fibrocytes in ESCC patients (*n* = 17) and healthy donors (*n* = 8) by FCM. Fibrocytes were identified by double-staining for FGFR2 and indicated fibrocyte markers. Horizontal lines represent the median. The whiskers extend to the highest values excluding outliers and extremes. ***, *P* < 0.001. **C.** The proportions of FGFR2^+^ fibrocyte progenitors (FGFR2^+^CD34^+^ or FGFR2^+^CD45^+^ cells) in BM were measured by FCM (upper panel) at 4 weeks after s.c. injecting KYSE30 or PBS. The data are represented as mean ± SD on 3 independent samples. **, *P* < 0.01. FGFR2^+^ fibrocyte precursors in the same samples were also detected by IF (lower panel). Double positive (FGFR2^+^CD34^+^ or FGFR2^+^CD45^+^) cells are indicated by yellow arrows. Selected regions are magnified. **D.** Luciferase-expressing human fibrocytes (hFbcs) were subjected to the in vivo chemotaxis assays in tumor-bearing mice (#5 and #6; s.c. injected with GFP-expressing KYSE30) or control mice (#1 and #2; s.c. injected with PBS). hFbcs distribution (upper panel) and tumor burden (lower panel) were determined at 24 h after i.v. injection of hFbcs. Tumor-bearing mice with PBS i.v. injection (#3 and #4) were used to discard non-specific background signals. Three mice will be evaluated for each condition. **E.** IHC staining demonstrated the presence of FGFR2^+^ cells or exogenous cells (Luciferase^+^ cells) in the xenografts obtained from the nude mice in in vivo chemotaxis assays. Abbreviations: HSC, hematopoietic stem cell; FCM, flow cytometry; hFbc, human fibrocyte; IHC, immunohistochemistry; IF, immunofluorescence; SD, standard deviation

Circulating cells gated in P2 can be divided into 3 main subsets according to the expression of specific cell-surface markers: CD14^+^CD16^+^ cells are classical monocytes, CD90^+^CD105^+^ cells are identified as MSCs, whereas CD34^+^CD45^+^Collagen I^+^ cells are fibrocytes that are originated from HSCs and thought to represent the precursors of fibroblast [19, 20]. To determine FGFR2^+^ circulating cells belong to which subset, peripheral blood samples from tumor implantation and resection mice were characterized by two-color FCM. Isolated leukocytes were double-stained for FGFR2 in combination with monocyte markers (CD14 and CD16), MSC markers (CD90) and fibrocyte markers (CD34, CD45 and Collagen I), respectively. As shown in Fig. 2A, both FGFR2^+^CD14^+^ and FGFR2^+^CD16^+^ cells were barely detectable (< 1%) in P2-gated cells. FGFR2^+^CD90^+^ cells were increased slightly after tumor resection (all *P* < 0.05) but not after ESCC implantation (all *P* ≥ 0.05). While, the FGFR2^+^ cells co-express CD34, CD45 or Collagen I was elevated in active tumor-bearing mice when compared with indolent tumor-bearing or PBS-treated mice (*P* < 0.01 for CD34^+^ FGFR2^+^ cells; *P* < 0.01 for CD45^+^ FGFR2^+^ cells; *P* < 0.05 for Col I^+^ FGFR2^+^ cells), but returned to near-basal levels after active tumor removal. These results indicated that FGFR2^+^ circulating cells induced by active tumor xenografts were murine fibrocytes.

We next compared the proportions of FGFR2 and fibrocyte marker double-positive circulating cells between ESCC patients and healthy volunteers. The cells co-expressing FGFR2 and CD34/CD45/Collagen I were present at significantly higher levels in patients with ESCC (all *P* < 0.001; Fig. 2B). These findings supported that tumor-induced FGFR2^+^ fibrocytes also exist in ESCC patients.

To confirm the HSC origin of the FGFR2^+^ fibrocytes, FCM analysis and IF staining were performed on BM cells obtained from KYSE30 tumor-bearing mice. The result found that progenitors of FGFR2^+^ fibrocytes (FGFR2^+^CD34^+^CD45^+^ cells) in BM were significantly increased in KYSE30 tumor-bearing mice when compared with non-tumor-bearing mice (*P* < 0.01; Fig. 2C). This observation investigated that FGFR2^+^ fibrocytes induced by ESCC were originated from HSC.

### ESCC recruits FGFR2^+^ fibrocytes into tumor mass

We next examined whether ESCC cells were able to recruit FGFR2^+^ fibrocytes using an in vivo chemotaxis model. FGFR2^+^ fibrocytes were isolated from either ESCC patients or KYSE30 tumor-bearing mice (cell purity > 94%; Fig. S1), transfected with lentiviral vector carrying Luciferase gene and injected into the tail vein of KYSE30 (labeled with GFP) tumor-bearing mice. Non tumor-bearing mice intravenously (i.v.) injected with fibrocytes and tumor-bearing mice i.v. injected with PBS were used as controls. Twenty-four hours after i.v. injection, fibrocyte distribution (luminescent signals) and tumor burden (fluorescence signals) were detected by in vivo imaging system, respectively. From Fig. 2D and Fig. S2A, we found that both human and murine fibrocytes distributed all over the bodies of the non-tumor-bearing mice. However, in tumor-bearing mice, they were accumulated in tumor regions, demonstrating that FGFR2^+^ fibrocytes could be recruited into tumor mass in vivo. The exogenous FGFR2^+^ cells in xenografts were further confirmed by IHC staining with FGFR2 and Luciferase antibodies (Fig. 2E and Fig. S2B), which could not be observed in other organs (Fig. S2C).

### FGFR2^+^ fibrocytes differentiate into functional CAFs through direct cell–cell contact with ESCC cells

**Fig. 3:**
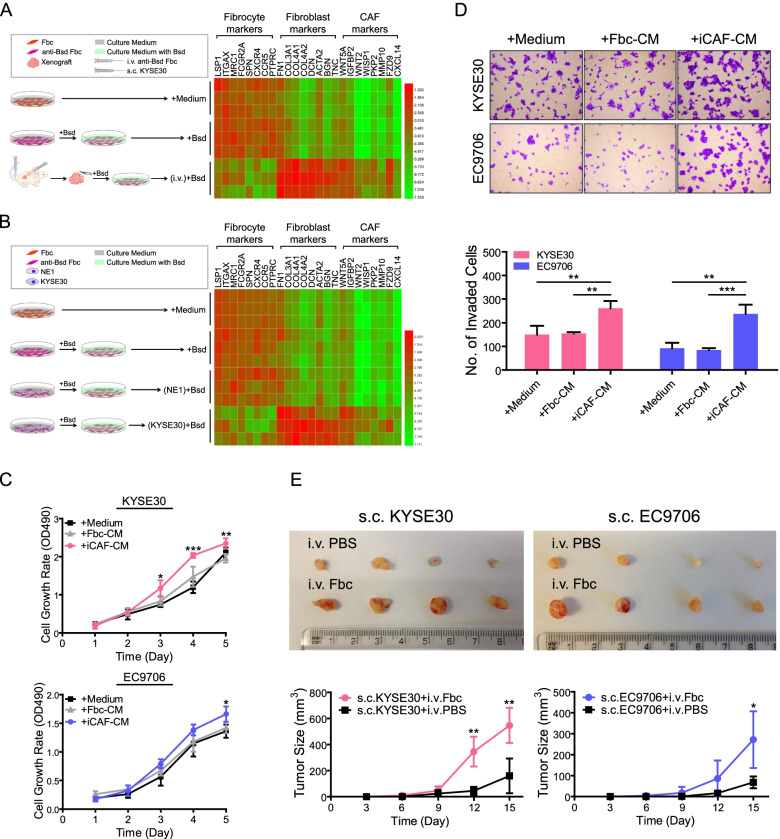
ESCC cells trigger differentiation of fibrocytes into mature CAFs through direct cell–cell contact. **A.** Expression of indicated marker genes in Bsd resistant-Fbcs direct contacted with KYSE30 in vivo [(i.v.) + Bsd] were determined by qPCR and summarized in heat map. The cells cultured with normal culture medium (+ Medium) or medium containing Bsd (+ Bsd) were used as controls. The experiment was repeated three times. **B.** Expression of indicated marker genes in Bsd resistant-Fbcs direct co-cultured with KYSE30 [(KYSE30) + Bsd] were determined by qPCR and summarized in heat map. The cells cultured with normal culture medium (+ Medium) or medium containing Bsd (+ Bsd) or direct co-cultured with NE1 [(NE1) + Bsd] were used as controls. The experiment was repeated three times. Schematic diagrams in A-B showing the experimental procedures of differentiation assays. Color intensity of heat map represents log10(Relative expression) within each measurement. **C.** The effect of normal culture medium (+ Medium), CM from Fbcs (+ Fbc-CM) or CM from iCAFs (+ iCAF-CM) on the growth of ESCC cells (KYSE30 and EC9706) was compared by XTT assay. The data are represented as mean ± SD on 3 independent experiments. *, *P* < 0.05; **, *P* < 0.01; ***, *P* < 0.001. **D.** Representative images showing the invasion of ESCC cells (KYSE30 and EC9706) cultured in the normal culture medium, Fbc-CM or iCAF-CM. Number of invaded cells was quantified in the lower panel. The data are represented as mean ± SD on 3 independent experiments. **, *P* < 0.01; ***, *P* < 0.001. **E**. Representative images of xenograft tumors (generated by KYSE30 and EC9706) harvested from nude mice with Fbc or PBS i.v. injection. Tumor growth curves are summarized in the bottom panel. The data are represented as mean ± SD in 4 mice. *, *P* < 0.05; **, *P* < 0.01. Abbreviations: CAF, cancer-associated fibroblast; Bsd, blasticidin; Fbc, fibrocyte; qPCR, semi-quantitative PCR; CM, conditioned medium; iCAF, induced-cancer-associated fibroblast

Given the evidence that normal fibrocytes can further differentiate into mature fibroblasts after homing into injured sites [19, 20], it is natural to wonder if FGFR2^+^ fibrocytes can differentiate into CAFs after arriving in ESCC tumor mass. We evaluated the differentiation capability of FGFR2^+^ fibrocytes here, using an in vivo model. In brief, we isolated FGFR2^+^ fibrocytes from three ESCC patients and transformed these cells into blasticidin (Bsd) resistant-fibrocytes by transfection of Bsd resistance genes. The transfectants were then injected into ESCC tumor-bearing mice through tail vein. One week later, exogenous cells were isolated from tumor xenografts via Bsd selection. Their cell identities were accessed by measuring the expression of fibrocyte, fibroblast and CAF markers using qPCR (marker gene sets were selected based on previous studies [10, 19]). As shown in Fig. 3A, the expression levels of fibroblast and CAF markers were increased, while fibrocyte markers were decreased in the cells isolated from xenografts as compared to parental or Bsd selected fibrocytes, indicating that FGFR2^+^ fibrocytes can differentiate into CAFs under ESCC-microenvironmental condition.

To determine whether fibrocyte-to-CAF differentiation is trigger by paracrine factors of ESCC cells, we initially cultured fibrocytes with conditioned medium (CM) of KYSE30. After a culture period of 48 h, we failed to observe any significant difference between KYSE30-CM-treated cells and control cells (the cells treated with either normal culture medium or CM of normal esophageal epithelial cell line NE1) (Fig. S3A). Similar results were observed in the experiments using transwell co-culture systems which only permitted cellular intercommunication via soluble factors (Fig. S3B), implying that paracrine factors produced by ESCC cells can not trigger the fibrocyte-to-CAF differentiation. Next, we tested whether direct cell–cell contact with ESCC cells can trigger CAF differentiation by direct co-culturing Bsd resistant-fibrocytes with KYSE30. Dramatic changes were observed in fibrocyte-KYSE30 co-cultures when compared to control monocultures or fibrocyte-NE1 co-cultures. The fibrocytes contracted with KYSE30 displayed similar expression patterns with CAFs (Fig. 3B). To confirm if these induced CAFs (iCAFs) obtained functional abilities, we tested proliferation and invasion of ESCC cells (KYSE30 and EC9706) exposed to the CM of iCAF. Results showed that both cell growth rates and invaded cell numbers of ESCC cells treated with iCAF-CM were increased when compared to control fibrocyte-CM or normal medium (Fig. 3C-D). Consistently, tumorigenicity assay demonstrated that iCAFs could promote tumor formation in nude mice (Fig. 3E).

### ESCC cells stimulate the expansion of FGFR2.^+^ fibrocyte progenitors via secreting FGF2

**Fig. 4:**
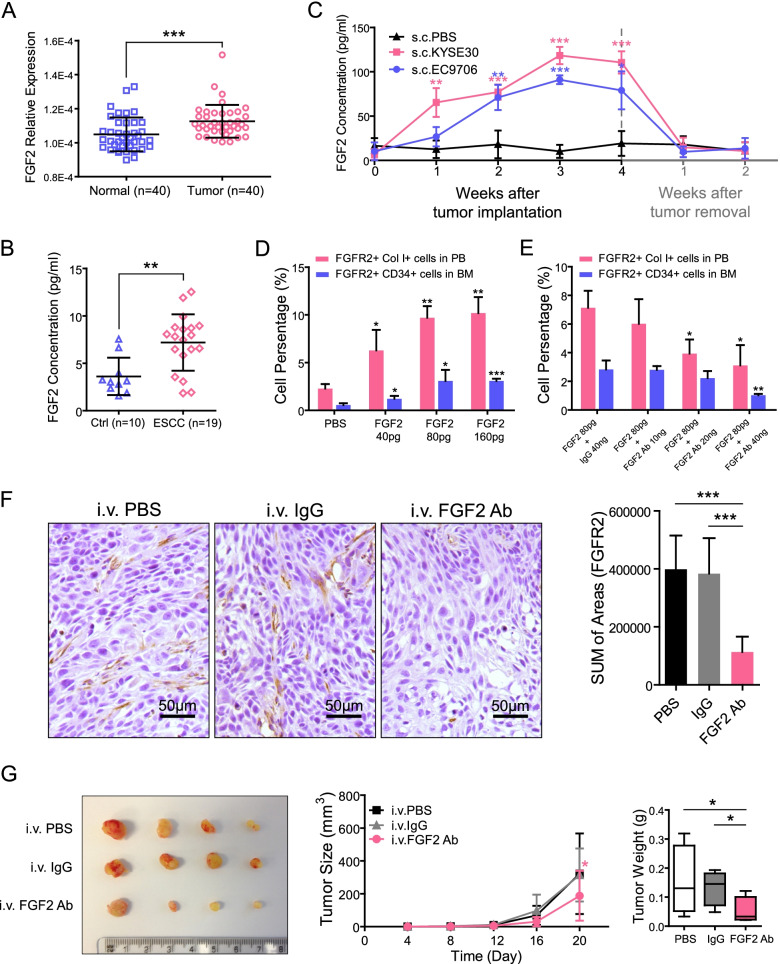
ESCC cells induce fibrocyte precursor expansion through FGF2. **A.** Relative expression of FGF2 in primary ESCCs (*n* = 40) and adjacent normal tissues (*n* = 40) were detected by qPCR. Horizontal lines represent the median. The whiskers extend to the highest values excluding outliers and extremes. ***, *P* < 0.001. **B.** FGF2 levels in serum samples from ESCC patients (*n* = 19) and healthy donors (*n* = 10) were measured by ELISA. Horizontal lines represent the median. The whiskers extend to the highest values excluding outliers and extremes. **, *P* < 0.01. **C.** Serum FGF2 levels were monitored by ELISA at a series of indicated time points after tumor implantation with ESCC cells or tumor resection. The data are represented as mean ± SD on 5 independent samples. **, *P* < 0.01; ***, *P* < 0.001. **D.** FCM was used to detect the proportions of fibrocyte precursors (FGFR2^+^CD34^+^ cells) and circulating fibrocytes (FGFR2^+^Col I^+^ cells) in nude mice treated with 3 different doses of FGF2 recombinant protein. The data are represented as mean ± SD on 4 independent samples. *, *P* < 0.05; **, *P* < 0.01; ***, *P* < 0.001. **E.** FCM was used to detect the proportions of fibrocyte precursors and circulating fibrocytes in nude mice treated with 80 pg FGF2 recombinant protein and increasing concentrations of FGF2 neutralizing antibody. The data are represented as mean ± SD on 4 independent samples. *, *P* < 0.05; **, *P* < 0.01. **F**. Representative IHC images of FGFR2^+^ cells in the KYSE30 xenografts obtained from the nude mice treated with FGF2 neutralizing antibody, control IgG or PBS. The immunostaining area of FGFR2 was summarized in the right panel. The data are represented as mean ± SD in 4 mice. ***, *P* < 0.001. **G**. The therapeutic effect of FGF2 neutralizing antibody was evaluated on KYSE30 tumor-bearing mice. Mice were treated intravenously everyday with FGF2 neutralizing antibody (4 μg/kg), control IgG or PBS. Xenografts were harvested at day 20 (left panel). Tumor size and tumor weight was summarized in the middle panel and right panel. The data are represented as mean ± SD in 4 mice. *, *P* < 0.05. Abbreviations: ESCC, esophageal squamous cell carcinoma; qPCR, semi-quantitative PCR; ELISA, enzyme-linked immunosorbent assay; IHC, immunohistochemistry

As FGF2/FGFR2 axis has been implicated in activation and expansion of BM progenitors [21], coupled with the fact that FGF2 expression was found to be upregulated in ESCC [22, 23], we wonder if ESCC cells can stimulate the expansion of FGFR2^+^ fibrocyte progenitors in BM via secreting FGF2. Our qPCR results confirmed that the average expression level of FGF2 was significantly higher in ESCC specimens than in their adjacent non-tumor counterparts (*P* < 0.001; Fig. 4A). ELISA assay also found that ESCC patients had higher FGF2 level in serum as compared with healthy individuals (*P* < 0.01; Fig. 4B). To more accurately determine whether circulating FGF2 in ESCC was predominantly produced by cancer cells, we subcutaneously injected KYSE30 or EC9706 cells into nude mice and monitored their FGF2 levels in serum by ELISA assay. In tumor-bearing mice, serum FGF2 increased steadily from baseline levels (11.84 ± 10.70 pg/mL) to the peak (104.72 ± 16.55 pg/mL) within 3 weeks after tumor implantation (*P* < 0.001), and declined immediately after tumor removal (Fig. 4C), confirming that serum FGF2 was mainly secreted by ESCC cells.

Next, we investigated whether FGF2 was responsible for inducing the expansion of FGFR2^+^ fibrocyte progenitors. Results showed that i.v. injection of recombinant FGF2 protein in nude mice led to a dose-dependent increase of FGFR2^+^ fibrocyte progenitors (FGFR2^+^CD34^+^ cells) in BM and circulating fibrocytes (FGFR2^+^Col I^+^ cells) in peripheral blood as compared with PBS-treated mice (Fig. 4D). While, blocking FGF2 with neutralizing antibodies resulted in ~ 50% reduction in these two types of cells compared with controls (Fig. 4E), supporting that FGF2/FGFR2 axis contributes to the expansion of fibrocyte progenitors. To extend these findings, we treated ESCC tumor-bearing mice (induced by either KYSE30 or EC9706 cells) with FGF2 neutralizing antibody for 20 days and analyzed the effects on tumorigenesis and infiltrated FGFR2^+^ CAFs. FGF2 blocking resulted in a marked reduction in FGFR2^+^ CAFs of the treated mice (*P* < 0.001, Fig. 4F; *P* < 0.01, Fig. S4A). Furthermore, it inhibited the tumorigenesis and reduced cancer progression when compared to isotype or PBS control mice (*P* < 0.05 for tumor size and tumor weight, Fig. 4G and Fig. S4B).

### RNA-seq reveals potential molecular mechanisms associated with the recruitment and differentiation of FGFR2^+^ fibrocytes

**Fig. 5:**
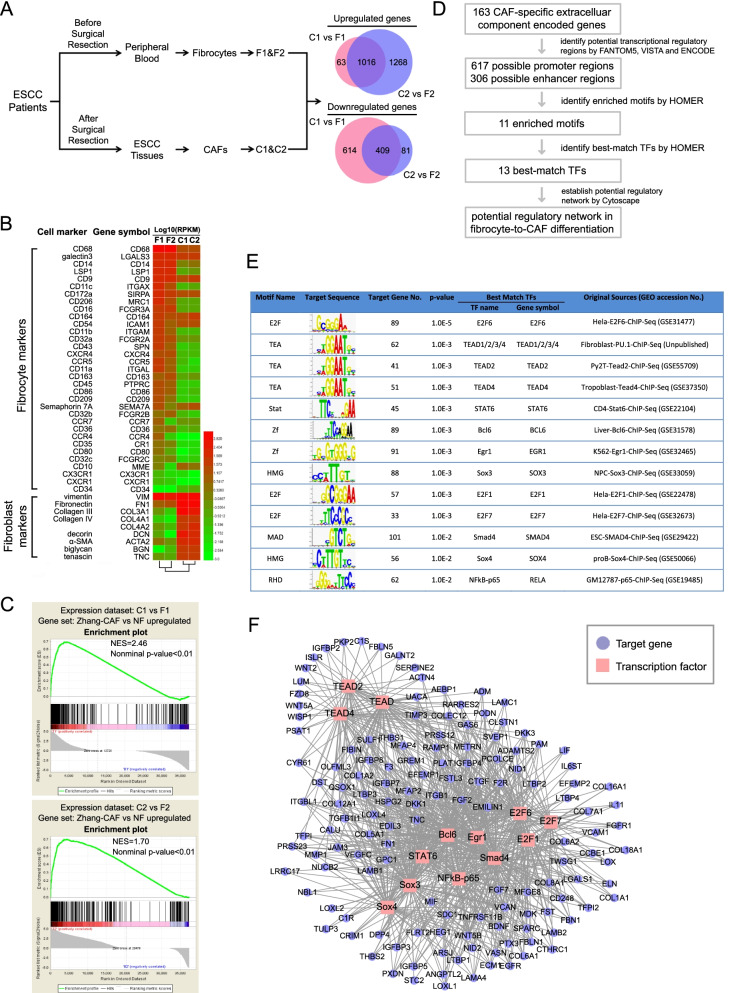
RNA-seq reveals potential molecular mechanisms involved in the differentiation of FGFR2^+^ fibrocytes. **A**. The expression signatures of FGFR2^+^ fibrocytes and CAFs collected from ESCC patients were analyzed by RNA-seq. The process about sample collection was summarized in the flowchart. The Venn diagram showing the number of overlapping transcripts detected in each context. **B.** The expression of indicated marker genes in fibrocytes (F1 and F2) and CAFs (C1 and C2) were summarized in heat map. Color intensity of heat map represents log10(RPKM) within each measurement. **C.** The correlation between our RNA-seq data (C1 vs F1 and C2 vs F2) and genes upregulated in CAFs versus Normal fibroblasts (Gene set: Zhang-CAF vs NF upregulated) were determined by GSEA analysis. The NES and the nominal *P*-value are indicated. **D.** Schematic diagram of regulatory network analysis for identifying key transcription factors associated with fibrocyte differentiation. **E.** Motif enrichment analysis was used to determine which transcription factors control the transcription of gene set associated with fibrocyte differentiation. The results were summarized in the table. (*P*-value report threshold: 0.01). **F.** The regulatory module of 13 predicted transcription factors and their target genes. Abbreviations: ESCC, esophageal squamous cell carcinoma; CAF, cancer-associated fibroblast; RPKM, reads per gene per kilobase exon per million mapped reads; GSEA, gene set enrichment analysis; NES, normalized enrichment score; NF, normal fibroblast; RNA-seq, RNA-sequencing

To obtain an accurate overview of changes occurring during fibrocyte-to-CAF differentiation in ESCC, we isolated circulating FGFR2^+^ fibrocytes and their paired FGFR2^+^ CAFs from 9 ESCC patients and compared their gene expression profiles by high-throughput RNA-seq (Gene Expression Omnibus accession number GSE103111). Samples (total RNA) were pooled into two groups: group 1 combined 5 samples of fibrocytes (F1) and corresponding paired CAFs (C1), while group 2 combined 4 samples of fibrocytes (F2) and marched CAFs (C2). The library statistics of RNA-seq was summarized in Table S7. On average, about 55.08 million reads were obtained after eliminating low quality reads and 52.76 million reads (95.79%) were aligned to the human mRNA reference sequences. Transcripts corresponding to a total of 1,016 and 409 genes were found to be upregulated or downregulated at least two-fold in the combined set of two CAF sample pools, respectively (Fig. 5A). Nearly all fibroblast markers were upregulated while fibrocyte markers were downregulated in CAFs (marker gene sets were obtained from Reilkoff et al*.* [19]; Fig. 5B). Moreover, GSEA confirmed that FGFR2^+^ fibrocytes also acquired CAF specific gene signatures during differentiation, as a set of genes known to be upregulated in CAFs when compared with their paired normal fibroblasts was also significantly elevated in our RNA-seq data (the gene set used in this analysis was obtained from Zhang et al*.* [10]; Fig. 5C). Taken together, these observations demonstrate that our RNA-seq data is of high quality and suitable for further analysis.

To characterize the functional consequences of gene expression changes associated with fibrocyte-to-CAF differentiation, we performed GO enrichment analysis. As shown in Fig. S5A, most of the upregulated genes in CAFs encoded ECM and secreted proteins. They were mainly involved in system development, biological adhesion and cellular component organization. Moreover, downregulated genes were characterized with functions of receptor activity, cytokine binding and chemokine binding. They were mostly involved in regulation of immune system process, immune response and response to stress or stimulus. Using pathway analysis based on KEGG database, we found that significant signaling pathways of upregulated genes included ECM-receptor interaction and Hippo pathway. However, downregulated genes enriched in the pathways related to chemokine signaling, cytokine-cytokine receptor interaction and transendothelial migration (Fig. S5B). All these data suggest that FGFR2^+^ fibrocytes simultaneously gain the abilities of ECM production and stroma construction; enhance the interactions with other cells in tumor mass and activate differentiation associated signaling pathways, such as Hippo pathway, upon differentiation into CAFs. However, they gradually lose their abilities for immune response, cell trafficking and transendothelial migration through decreased the expression of specific cytokines and chemokine receptors.

It is particularly noteworthy that 6 chemokine receptors in GO term “chemokine binding”, including CXCR4, CCR5, CCR1, CCRL2, CMKLR1 and A2M, are specific expressed in FGFR2^+^ fibrocytes before arriving in tumor sites suggesting their possible roles in fibrocyte recruitment. Of these, CXCR4, was found to be essential for fibrocyte recruitment in asthma [24], whereas its ligand CXCL12 was reported to be highly expressed in ESCC tissues [25]. Therefore, we postulated that CXCL12/CXCR4 axis could play a key role in the recruitment of FGFR2^+^ fibrocytes in ESCC.

Producing a variety of CAF-specific extracelluar factors is a key function of CAFs. To understand how FGFR2^+^ fibrocytes acquire this function upon differentiation into CAFs, we tried to identify potential master regulators of CAF-specific extracelluar factors by motif enrichment analysis (Fig. 5D). The gene set used in this analysis containing 163 extracelluar component encoded genes which upregulated in CAFs when compared to both fibrocytes and normal fibroblasts (Table S8). Using FANTOM5, VISTA and ENCODE, a total of 617 promoter regions and 306 enhancer regions were identified. A motif finder algorithm, HOMER, was then utilized to identify motifs localized in these possible transcriptional regulatory regions. Top 11 scoring motifs (*P* < 0.01) and their best-match transcription factors were listed in Fig. 5E. Additionally, the predicted transcriptional regulatory networks were constructed by Cytoscape (Fig. 5F and Table S6). From the results, we observed a binding motif for TEAD transcription factors as one of the most enriched regulatory elements in the promoters/enhancers of CAF-specific extracelluar factor encoded genes. This finding suggested a potential role of TEAD family in priming FGFR2^+^ fibrocyte to CAF differentiation.

### ESCC cells recruit FGFR2^+^ fibrocytes mainly through the CXCL12/CXCR4 axis

**Fig. 6:**
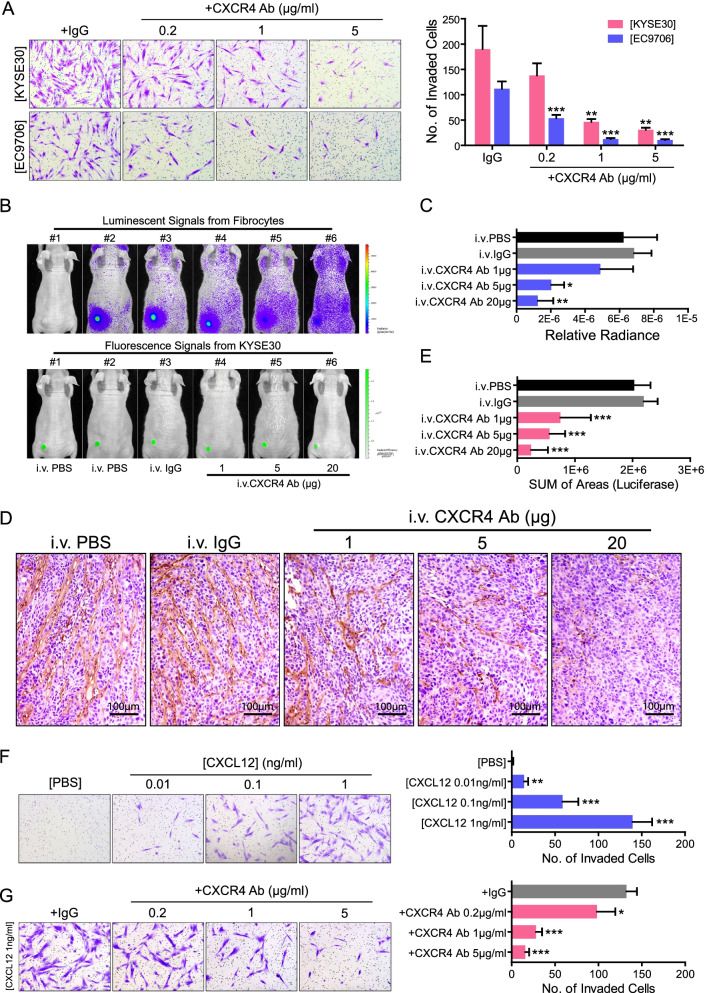
CXCL12/CXCR4 is involved in the recruitment of FGFR2^+^ fibrocytes to ESCC tumor mass. **A.** The blocking effect of CXCR4 neutralizing antibody on ESCC-induced fibrocyte migration was evaluated by in vitro chemotaxis assay. hFbcs were isolated from ESCC patients and treated by increasing concentrations of CXCR4 neutralizing antibody or control IgG for 24 h. Indicated cell lines ([KYSE30] or [EC9706]) were used as chemotactic stimulus. Representative images of hFbcs invaded through matrigel are shown in the left panel. Number of invaded hFbcs was quantified in the right panel. The data are represented as mean ± SD on 3 independent experiments. **, *P* < 0.01; ***, *P* < 0.001. **B-C.** The blocking effect of CXCR4 neutralizing antibody on the KYSE30-induced chemotaxis was evaluated by in vivo chemotaxis assay. 1 h after i.v. injection with Luciferase-expressing hFbcs, tumor-bearing mice (generated by GFP-expressing KYSE30) were treated with increasing doses of CXCR4 neutralizing antibody (#4, #5 and #6), control IgG (#3) or PBS (#2). hFbcs distribution (upper panel in B) and tumor burden (lower panel in B) were determined at 24 h after CXCR4 neutralizing antibody injection. Tumor-bearing mice treated with PBS (#1) were used to discard non-specific background signals. The relative radiance was summarized in C. The data are represented as mean ± SD on 4 independent experiments. *, *P* < 0.05; **, *P* < 0.01. **D-E.** Representative IHC images of exogenous cells (Luciferase^+^ cells) in KYSE30 xenografts obtained from the nude mice treated with CXCR4 neutralizing antibody, control IgG or PBS. The immunostaining area of Luciferase was summarized in E. The data are represented as mean ± SD in 4 mice. ***, *P* < 0.001. **F.** CXCL12-induced chemotaxis in hFbcs was measured using transwell system. Increasing concentrations of CXCL12 recombinant protein or PBS were used as chemotactic stimulus. Representative images of hFbcs invaded through matrigel are shown in the left panel. Number of invaded hFbcs was quantified in the right panel. The data are represented as mean ± SD on 3 independent experiments. **, *P* < 0.01; ***, *P* < 0.001. **G.** The blocking effect of CXCR4 neutralizing antibody on CXCL12-induced hFbc migration was evaluated by in vitro chemotaxis assay. hFbcs were treated by increasing concentrations of CXCR4 neutralizing antibody or control IgG for 12 h. CXCL12 were used as chemotactic stimulus. Representative images of hFbcs invaded through matrigel are shown in the left panel. Number of invaded hFbcs was quantified in the right panel. The data are represented as mean ± SD on 3 independent experiments. *, *P* < 0.05; ***, *P* < 0.001. Abbreviations: hFbc, human fibrocyte; ESCC, esophageal squamous cell carcinoma; i.v., intravenous

To test whether CXCL12/CXCR4 axis plays a critical role in the recruitment of FGFR2^+^ fibrocytes, we first assessed the chemotactic ability of fibrocytes after CXCR4 knockdown by specific shRNAs (Fig. S6A). During the experiments, KYSE30 and EC9706 cells were seeded onto the lower chambers to serve as chemotactic stimuli. By calculating the number of invaded fibrocytes, we found that the chemotactic behaviors were suppressed in CXCR4-knockdown cells as compared to scramble shRNA-treated cells (Fig. S6B). Furthermore, this chemotaxis phenotype induced by ESCC cells could be blocked by CXCR4 neutralizing antibody (Fig. 6A).

The blocking effect of CXCR4 neutralizing antibody on the ESCC-induced chemotaxis was further evaluated in vivo. One hour after i.v. injection with Luciferase-expressing fibrocytes, ESCC tumor-bearing mice were treated with CXCR4 neutralizing antibody, PBS or IgG, respectively. After 24 h, fibrocytes distribution and tumor burden were determined (Fig. 6B-C and Fig. S7A-B). The treatments were continued until the xenografts were harvested one week after the initial injection. The infiltration of Luciferase-expressing fibrocytes in xenografts were then detected by IHC and quantified by Image-Pro Plus 6.0 software (Fig. 6D-E and Fig. S7C-D). Consistently, CXCR4 neutralizing antibody could suppress fibrocyte accumulation in ESCC tumors.

To determine whether CXCL12 participate in FGFR2^+^ fibrocyte recruitment, we first established whether a CXCL12 gradient existed between ESCC tumor mass and peripheral blood by ELISA. As shown in Fig. S6C, CXCL12 concentrations were significantly higher in ESCC tumor homogenates than in plasmas of ESCC patients (*P* = 0.038). In contrast, no CXCL12 gradient was observed between homogenates of non-tumor tissues and plasmas (*P* = 0.578). The abilities of FGFR2^+^ fibrocytes migration along the CXCL12 gradients were then determined by transwell invasion assay. The results showed that CXCL12 could chemoattract the fibrocytes in transwell coculture systems (Fig. 6F), which could be effectively suppressed by CXCR4 neutralizing antibody in a dose-dependent manner (Fig. 6G). Taken together, all these data reveal that ESCC cells recruit FGFR2^+^ fibrocytes mainly through the CXCL12/CXCR4 axis.

### Activation of YAP-TEAD transcriptional complex is essential for FGFR2^+^ fibrocyte-to-CAF Differentiation

**Fig. 7:**
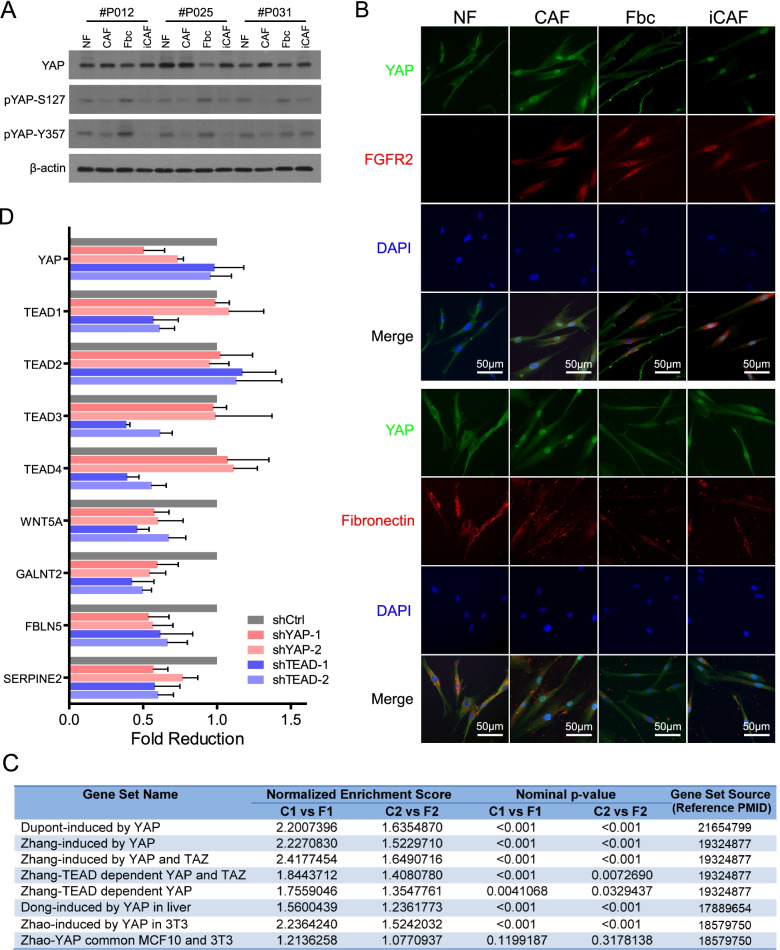
YAP-TEAD complex activation is involved in FGFR2^+^ fibrocyte differentiation. **A**. The expression levels of total YAP and inactive YAP (pYAP-S127/Y357) were detected in NF, CAF, Fbc and iCAF from 3 ESCC patients by western blots. β-actin was used as loading control. **B.** Representative IF images of subcellular localization of YAP in NF (FGFR2^−^ Fibronectin^strong^), CAF (FGFR2^+^Fibronectin^strong^), Fbc (FGFR2^+^Fibronectin^weak^) and iCAF (FGFR2^+^Fibronectin^strong^). Nuclei were counterstained with DAPI. **C**. GSEA analysis of multiple TEAD and YAP/TAZ gene sets was performed on our RNA-seq data (C1 vs F1 and C2 vs F2). The normalized enrichment score, nominal p-value and the sources of gene sets are summarized in the table. **D**. Expression of indicated genes in YAP/TEAD knockdown CAFs were measured by qPCR. The data are represented as mean ± SD of the fold reduction compared to scramble control (shCtrl). The experiment was repeated three times. Abbreviations: GSEA, gene set enrichment analysis; NF, normal fibroblast; CAF, cancer-associated fibroblast; Fbc, fibrocyte; iCAF, induced-cancer-associated fibroblast; qPCR, semi-quantitative PCR; IF, immunofluorescence; ESCC, esophageal squamous cell; Ctrl, control

Transcription factors in TEAD family (TEAD 1/2/3/4) were predicted as master regulators in FGFR2^+^ fibrocyte-to-CAF differentiation by our motif enrichment analysis (Fig. 5E-F). Their transcriptional activity is regulated by Hippo pathway. Activation of this pathway phosphorylates the YAP/TAZ complex, a transcription co-activator complex of TEADs. Phosphorylated YAP proteins bind to 14–3-3ε in the cytoplasm, thereby hindering their nuclear import and thus blocking their function. When dephosphorylated, YAP/TAZ complexes translocate into the nucleus and interact with TEADs to induce expression of genes that control cell-fate decisions [26]. Here we found that the inactive forms of YAP (pYAP-S127 and pYAP-Y357) were reduced in CAFs and iCAFs as compared with FGFR2^+^ fibrocytes and normal fibroblasts (Fig. 7A). Additionally, YAP was predominantly cytoplasmic in FGFR2^+^ fibrocytes and normal fibroblasts but accumulated in the nucleus in CAFs and iCAFs (Fig. 7B), suggesting that the nuclear localization of YAP is a universal feature of CAFs differentiation in ESCC. Furthermore, GSEA analysis revealed that the genes induced by TEADs and/or YAP/TAZ showed significant overlap with the genes upregulated in our RNA-seq data (Fig. 7C), suggesting that YAP-TEAD transcriptional complex is activated upon fibrocyte-to-CAF differentiation.

To further determine the regulatory role of YAP-TEAD complex, we separately knocked down YAP and TEADs in CAFs by shRNAs and evaluated the expression levels of 4 genes (WNT5A, GALNT2, FBLN5 and SERPINE2) that our motif enrichment analysis had indicated as TEAD target genes (Fig. 5F). As shown in Fig. 7D, the shRNAs of TEADs were only able to knockdown TEAD1, TEAD3, and TEAD4 concurrently but not TEAD2. Nevertheless, they still blocked the expression of TEAD target genes identified by our motif enrichment analysis. Likewise, silencing YAP expression could significantly suppress the expression of TEAD downstream targets, demonstrating that YAP-TEAD complex activation is required for FGFR2^+^ fibrocyte-to-CAF differentiation.

## Discussion

**Fig. 8:**
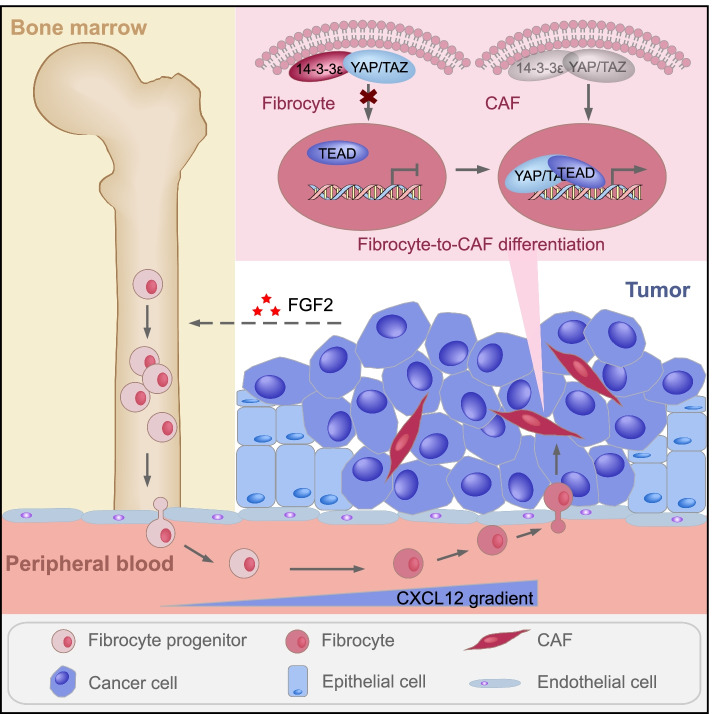
A schema for illustrating the mechanism about how FGFR2^+^ CAF progenitors mobilize from BM, recruit into tumor mass and differentiate into CAFs under the guidance of tumor cells in ESCC. Schematic drawing those FGFR2^+^ CAF progenitors in BM can be mobilized by cancer-secreted FGF2 and recruited into tumor sites via the CXCL12/CXCR4 axis. Moreover, they differentiate into CAFs through the activation of YAP-TEAD complex which is trigger by directly contract with tumor cells**. **Abbreviations: CAF, cancer-associated fibroblast; BM, bone marrow; ESCC, esophageal squamous cell carcinoma

Although it is widely accepted that CAF is an ideal target for cancer treatment, insufficient understanding of the mechanisms underlying CAF generation have slowed down the developmental progress of cancer therapy. Here, we systematically characterized the interaction process between tumor and FGFR2^+^ CAFs in ESCC, including how CAF progenitors were mobilized from BM, recruited into tumor sites and differentiated into functional CAFs by cancer cells (Fig. 8). This work will be of great interest to the development of CAF-based therapies. The main problem in CAF generation is where they arise from. There are several theories regarding the CAF origin. Several kinds of cells are considered as possible predecessors of CAFs [5]. While, in esophageal cancer, BM-derived cells have been commonly regarded as a potential source of CAFs [16]. In the present study, we used FGFR2, a specific CAF marker in ESCC, as a tracer to study the origin of CAFs and further identified CAF progenitors in BM.

Adult BM contains two types of stem cells, HSCs and MSCs. HSCs are defined by their ability to repopulate the BM and blood lineages of lethally irradiated mice. The cells arise from HSCs are necessarily and specific express the CD34 antigen. On the other hand, MSCs are strongly positive for CD90 and CD105. They are defined by their ability to give rise to a variety of mesenchymal cell types including fibroblasts [27, 28]. Therefore, it is widely held that the BM source of CAFs is MSCs. The first evidence for this notion came from an in vitro study demonstrating that human BM-derived MSCs can differentiate into CAFs when treated with conditioned media from cancer cells [29]. Subsequently, a number of murine tumor models and studies in patients who received BM transplantations provided evidence that BM-derived MSCs can develop into CAFs in vivo [30, 31]. However, recent studies have raised the possibility that HSCs also give rise to fibroblasts. For instance, Ogawa et al*.* [32] carried out a series of tissue reconstitution studies by single HSCs and found that a large part of fibroblasts in many tissues are derived from HSCs. Using the same murine models, Abangan et al*.* [33] demonstrated an HSC origin for circulating fibroblast precursors. Moreover, LaRue et al*.* [34] demonstrated that HSCs were the main source of CAFs in melanoma-bearing mice by using single HSC transplantation. Using marker-based method, we found here that ESCC tumor burden in vivo could increase the HSC-derived CAF progenitors in BM (defined as FGFR2^+^CD34^+^CD45^+^ cells) and HSC-derived CAF precursors in peripheral circulation (defined as FGFR2^+^CD34^+^CD45^+^Col I^+^ cells). Furthermore, both in vitro and in vivo functional studies showed that HSC-derived fibrocytes preferentially migrated and differentiated into mature CAFs in response to ESCC, indicating that FGFR2^+^ CAFs in ESCC are of HSC origin.

HSC-derived mesenchymal progenitors belong to myeloid lineage [32]. Several studies of myelopoiesis show that FGFR2 is also typically expressed in myeloid progenitor cells [18, 35], which can be mobilized by FGF2 [36]. More importantly, FGF2 expression has been reported to significantly increase in ESCCs [22, 23]. Taking these into account, we designed a series of in vivo experiments to determine whether FGF2/FGFR2 axis contributes to the expansion of CAF progenitors in ESCC. The results demonstrated that FGF2 could increase FGFR2^+^ CAF progenitors in BM and FGFR2^+^ fibrocytes in peripheral circulation in a dose-dependent manner, while blocking FGF2 led to a marked reduce of FGFR2^+^ CAFs in tumor, suggesting that FGF2 is responsible for the mobilization and expansion of CAF progenitors.

Our next question is how FGFR2^+^ fibrocytes are recruited into tumor sites. According to the disease process and organ involved, circulating fibrocytes can use different chemokine ligand-receptor pairs for tissue homing. Human fibrocytes express several chemokine receptors, including CXCR4, CCR7, CCR5, and CCR3 [37–39], while, murine fibrocytes express CXCR4 and CCR7, but also CCR2 [38–40]. In our study, we found that the FGFR2^+^ fibrocytes isolated from both ESCC patients and ESCC tumor-bearing mice could be recruited into tumor mass under the stimulation of ESCC cells. In addition, our RNA-seq data show that 6 chemokine receptors including CXCR4, CCR5, CCR1, CCRL2, CMKLR1 and A2M are significantly upregulated in human FGFR2^+^ fibrocytes when compared to their matched CAFs, suggesting that these chemokine receptors may be responsible for the recruitment of FGFR2^+^ fibrocytes. CXCR4 was selected for further study because it expresses in both human and mouse fibrocytes. Furthermore, the overexpression of its ligand, CXCL12, in ESCC tissues creates the gradient required for trafficking of CXCR4^+^ cells [41]. Results of our in vitro and in vivo studies underscore the importance of chemokine-mediated fibrocyte influx in ESCC, and indicate that FGFR2^+^ fibrocytes are mainly recruited through the CXCL12/CXCR4 axis. Clinically, manipulation of CXCL12/CXCR4 axis-mediated responses may contribute to the inhibition of CAF infiltration in ESCC tumor mass.

Previous studies about normal wound healing suggest that fibrocyte differentiation is influenced by a complex profile of cytokines within the area of tissue injury. For example, the differentiation of fibrocytes into fibroblasts is augmented in the presence of TGF-β or endothelin-1, manifested by the expression of fibroblast marker fibronectin and collagen [37, 39, 42]. However, the molecular mechanisms by which fibrocytes differentiate into CAFs has never been investigated. This study is the first study focusing on this topic. We performed both in vitro and in vivo experiments to investigate how FGFR2^+^ fibrocytes were differentiated into CAFs using FGFR2^+^ fibrocytes isolated from peripheral blood of ESCC patients. The results show that the differentiation of fibrocytes into CAFs only occurs when they directly contact with ESCC cells. Our latter RNA-seq and motif enrichment analysis indicating that YAP-TEAD transcriptional complex activation is required for fibrocyte-to-CAF differentiation. Current studies have shown that YAP-TEAD complex activation was a consequence of several inter-related events, for example, rearrangement of actin cytoskeleton in response to the stiffness of ECM and disruption of tight junctions in cultured mammalian cells were associated with the elevation of YAP-TEAD activity [43–45]. Since increased matrix stiffness and tight junctions’ alterations are features of most epithelium-derived solid tumors, we propose that these features in ESCC can trigger FGFR2^+^ fibrocytes-to-CAFs differentiation by activating YAP-TEAD complex. This mechanism can be further employed to locally produce antitumor payloads in cellular therapy.

## Conclusions

This study demonstrated that: (1) HSC-derived FGFR2^+^ fibrocytes are the source of CAFs in ESCC. They can be mobilized from bone marrow, recruited into tumor sites and differentiated into functional CAFs by ESCC cells. (2) The expansion of CAF progenitors in bone marrow is stimulated by cancer-secreted FGF2. Abrogation CAF progenitor expansion by FGF2 inhibition can inhibit local growth and prevent distant metastasis of cancer cells. (3) ESCC cells recruit FGFR2^+^ fibrocytes mainly through CXCL12/CXCR4 axis. Disruption of the CXCL12/CXCR4 axis can reduce CAF infiltration. (4) FGFR2^+^ fibrocytes differentiate into CAFs mainly through the activation of YAP-TEAD complex which is trigger by directly contract with cancer cells. These exciting findings may lead to new avenues of CAF-targeted therapy for ESCC. They suggested that the circulating fibrocytes isolated from peripheral blood of ESCC patients are expected to become ideal cellular therapeutic tools, not only because they are easy to obtain and modify, but also because they are sufficient to homing into TME and target cancer cells via locally produce therapeutic payloads.

## Supplementary Information

**Additional file 1.Table S1. **Recombinant proteins andneutralizing antibodies used in this study.** Table S2.**Antibodies used in this study. **Table S3. **shRNAs used in this study. **Table S4.**Primers used in this study. **TableS5.** Gene Sets for GSEA analysis. **TableS6.** Motif enrichment analysis result of CAF-specific extracellular componentencoded genes. **Table S7.**Summary of sequencing statistics and mapping of reads for 2 paired FGFR2^+^fibrocyte and CAF sample pools. **Table S8.**Gene set of CAF-specific extracellular component used in motif enrichmentanalysis.

**Additional file 2.**  **Fig.S1 **Characterization of fibrocyte isolates.** A. **Fibrocyteisolates were analyzed by IF staining using anti-FGFR2 (green) andanti-Collagen I (red) antibodies. Nuclei were labeled with DAPI (blue).Representative images of IF and light microscopic view (Bright field) areshown. **B. **The purity of isolatedfibrocytes was determined by FCM with FGFR2 and CD34/CD45/Collagen I doublestaining. Abbreviations:IF, immunofluorescence; FCM, flow cytometry. **Fig.S2 **FGFR2^+^ circulating fibrocytes can be recruited into ESCC tumormass.** A. **Luciferase-expressing murine fibrocytes (mFbcs) weresubjected to the *in vivo* chemotaxisassays in tumor-bearing mice (#3; s.c. injection of GFP-expressing KYSE30) orcontrol mice (#1; s.c. injected with PBS). mFbcs distribution (upper panel) andtumor burden (lower panel) were determined respectively at 24 hours after i.v.injection of mFbcs. Tumor-bearing mice with PBS i.v. injection (#2) were usedto discard non-specific background signals. Three mice will be evaluated foreach condition.** B. **IHC stainingdemonstrates the presence of FGFR2^+^ cells or exogenous cells(Luciferase^+^ cells) in the xenograft tumors obtained from the nudemice in chemotaxis assays. **C.** IHCstaining demonstrates the presence of exogenous cells (Luciferase^+^cells) in indicated organs obtained from the nude mice in chemotaxis assays. Abbreviations:mFbc, murine fibrocyte; s.c., subcutaneous; i.v., intravenous; IHC,immunohistochemistry. **Fig.S3 **Paracrine factors produced by ESCC cells can not trigger the differentiationof fibrocytes.** A.** Expression of indicated marker genes inFbcs treated with CM of KYSE30 (+KYSE30-CM) were determined by qPCR andsummarized in heat map. The cells treated with normal culture medium (+Medium)or CM of NE1 (+NE1-CM) were used as controls. The experiment was repeated threetimes.** B**. Expression of indicatedmarker genes in Fbcs co-cultured with KYSE30 ([KYSE30]) using transwell systemswere determined by qPCR and summarized in heat map. The cells cultured withnormal culture medium ([-]) or indirect co-cultured with NE1 ([NE1]) were usedas controls. The experiment was repeated three times. Schematic diagrams in A-Bshowing the experimental procedures of differentiation assays. Color intensityof heat map represents log10 (Relative expression) within each measurement. Abbreviations:Fbc, fibrocyte; CM, conditioned medium; qPCR, semi-quantitative PCR. **Fig.S4 **Treatment with FGF2 neutralizing antibody reduces tumor growth in micemodel.** A.** The presence of FGFR2^+^ cells (leftpanel) in the EC9706 xenograft tumors obtained from the nude mice with FGF2neutralizing antibody/control IgG treatment were detected by IHC staining andrepresentative images are shown. The immunostaining area of FGFR2 wassummarized in the right panel. The data are represented as mean ± SD in 4 mice.**, *P* <0.01. **B.** Thetherapeutic effect of FGF2 neutralizing antibody was evaluated on EC9706xenograft-bearing mice. Mice were treated intravenously everyday with FGF2neutralizing antibody (4 μg/kg), or control IgG. Xenograft tumors wereharvested at day 20 (left panel). Tumor volume and tumor weight was summarizedin the middle panel and right panel. The data are represented as mean ± SD in 4mice. *, *P* <0.05. Abbreviations:IHC,immunohistochemistry. **Fig.S5 **GO and KEGG pathway enrichment analysis of differentially expressed genes.** A**.Indicated are the top 10 enriched GO terms associated with biological process,cellular component, and molecular function for all upregulated (the enrichment *P*-valueand gene number of each term were summarized in pink horizontal bar chart) anddownregulated genes (the enrichment *P*-value and gene number of each termwere summarized in blue horizontal bar chart). **B**. Indicated are the top 10 enriched KEGG pathways for allupregulated (the enrichment *P*-value and gene number of each term weresummarized in pink horizontal bar chart) and downregulated genes (theenrichment *P*-value and gene number of each term were summarized in bluehorizontal bar chart). **Fig.S6 **CXCL12/CXCR4 axis contributes to the recruitment of FGFR2^+^fibrocytes in ESCC.** A**. CXCR4 relative expression in CXCR4knockdown hFbcs (shCXCR4-1 and shCXCR4-2) or scramble-control hFbcs (shCtrl)were determined by qPCR. The data are represented as mean ± SD on 3 independentexperiments. *, *P* <0.05; **, *P* <0.01. **B.** CXCR4 knockdown hFbcs and control hFbcs were subjected to *in vitro* chemotaxis assays in thepresence of indicated cell lines ([KYSE30] or [EC9706]) as chemotacticstimulus. Representative images of hFbcs invaded through matrigel are shown inthe upper panel. Number of invaded hFbcs per 40× field was quantified in thelower panel. The data are represented as mean ± SD on 3 independent experiments.*, *P* <0.05; **, *P* <0.01; ***, *P* <0.001. **C.** CXCL12 concentrations in plasmasamples of ESCC patients (PB; *n* = 19), primary ESCC tissues (T; *n*= 21) and their corresponding non-tumor tissues (N; , *n* = 21) weredetected by ELISA. Horizontal lines represent the median. The whiskers extendto the highest values excluding outliers and extremes. *, *P* <0.05.Abbreviations:ESCC, esophageal squamous cell carcinoma; hFbc, human fibrocyte;Ctrl, control; qPCR, semi-quantitative PCR; ELISA, enzyme-linked immunosorbentassay. **Fig.S7 **CXCR4 neutralizing antibody inhibit the recruitment of FGFR2^+^fibrocytes to EC9706 xenograft tumors.** A-B**. The blockingeffect of CXCR4 neutralizing antibody on the ESCC-induced chemotaxis wasevaluated by *in vivo* chemotaxisassay. 1 hour after i.v. injection with Luciferase-expressing hFbcs, tumorbearing mice (generated by GFP-expressing EC9706) were treated with increasingdoses of CXCR4 neutralizing antibody (#3 and #4) or control IgG (#2). hFbcsdistribution (upper panel in A) and tumor burden (lower panel in A) weredetermined respectively at 24 hours after CXCR4 neutralizing antibodyinjection. Tumor-bearing mice treated with PBS (#1) were used to discardnon-specific background signals. The relative radiance was summarized inhistogram. The data are represented as mean ± SD on in 4 mice. *, *P*<0.05; **, *P* <0.01. **C-D**.Representative IHC images of exogenous cells (Luciferase^+^ cells) inEC9706 xenografts obtained from the nude mice treated with CXCR4 neutralizingantibody or control IgG. The immunostaining area of Luciferase was summarizedin D. The data are represented as mean ± SD in 4 mice. **, *P* <0.01;***, *P* <0.001. Abbreviations: ESCC, esophagealsquamous cell carcinoma; i.v., intravenous; hFbc, human fibrocyte; IHC,immunohistochemistry.

## Data Availability

The data that support the findings of this study are available from the corresponding author upon reasonable request. The high-throughput RNA-seq data in this study are available from Gene Expression Omnibus (accession number: GSE103111).

## Abbreviations

CAF: Cancer-associated fibroblast
TME: Tumor microenvironment
ECM: Extracellular matrix
ESCC: Esophageal squamous cell carcinoma
RNA-seq: RNA-sequencing
MSC: mesenchymal stem cell
HSC: hematopoietic stem cell
BM: bone marrow
IF: immunofluorescence
FCM: flow cytometry
NF: normal fibroblast
iCAF: induced-cancer-associated fibroblast
i.v.: intravenous
PBMC: peripheral blood mononuclear cell
s.c.: subcutaneous
IHC: immunohistochemistry
shRNA: short hairpin RNA
ROI: region of interest
qPCR: semi-quantitative PCR
ELISA: enzyme-linked immunosorbent assay
rRNA: ribosomal RNA
cDNA: complementary DNA
RPKM: reads per gene per kilobase exon per million mapped reads
GSEA: gene set enrichment analysis
Bsd: blasticidin
CM: conditioned medium
Fbc: fibrocyte
hFbc: human fibrocyte
mFbc: murine fibrocyte
