# Supplementary material for: Identification and characterization of FGFR2+ hematopoietic stem cell-derived fibrocytes as precursors of cancer-associated fibroblasts induced by esophageal squamous cell carcinoma
Source: J Exp Clin Cancer Res. 2022 Aug 8;41:240. doi: 10.1186/s13046-022-02435-w (PMC9358838; doi:10.1186/s13046-022-02435-w)
Supplement: Supplementary file 2 — Additional file 2. Fig.S1 Characterization of fibrocyte isolates. A. Fibrocyteisolates were analyzed by IF staining using anti-FGFR2 (green) andanti-Collagen I (red) antibodies. Nuclei were labeled with DAPI (blue).Representative images of IF and light microscopic view (Bright field) areshown. B. The purity of isolatedfibrocytes was determined by FCM with FGFR2 and CD34/CD45/Collagen I doublestaining. Abbreviations:IF, immunofluorescence; FCM, flow cytometry. Fig.S2 FGFR2+ circulating fibrocytes can be recruited into ESCC tumormass. A. Luciferase-expressing murine fibrocytes (mFbcs) weresubjected to the in vivo chemotaxisassays in tumor-bearing mice (#3; s.c. injection of GFP-expressing KYSE30) orcontrol mice (#1; s.c. injected with PBS). mFbcs distribution (upper panel) andtumor burden (lower panel) were determined respectively at 24 hours after i.v.injection of mFbcs. Tumor-bearing mice with PBS i.v. injection (#2) were usedto discard non-specific background signals. Three mice will be evaluated foreach condition. B. IHC stainingdemonstrates the presence of FGFR2+ cells or exogenous cells(Luciferase+ cells) in the xenograft tumors obtained from the nudemice in chemotaxis assays. C. IHCstaining demonstrates the presence of exogenous cells (Luciferase+cells) in indicated organs obtained from the nude mice in chemotaxis assays. Abbreviations:mFbc, murine fibrocyte; s.c., subcutaneous; i.v., intravenous; IHC,immunohistochemistry. Fig.S3 Paracrine factors produced by ESCC cells can not trigger the differentiationof fibrocytes. A. Expression of indicated marker genes inFbcs treated with CM of KYSE30 (+KYSE30-CM) were determined by qPCR andsummarized in heat map. The cells treated with normal culture medium (+Medium)or CM of NE1 (+NE1-CM) were used as controls. The experiment was repeated threetimes. B. Expression of indicatedmarker genes in Fbcs co-cultured with KYSE30 ([KYSE30]) using transwell systemswere determined by qPCR and summarized in heat map. The cells cultur [file 13046_2022_2435_MOESM2_ESM.docx]

**Supplementary materials**

**Identification and characterization of FGFR2^+^ hematopoietic stem cell-derived fibrocytes as precursors of cancer-associated fibroblasts induced by esophageal squamous cell carcinoma**

Haibo Qiu^1^, Xu Zhang^2^, Jiali Qi^3^, Jiangwen Zhang^4^, Yin Tong^4^, Lei Li^5^, Li Fu^6^, Yan-Ru Qin^7^, Xinyuan Guan^3,*^, Liyi Zhang^3,*^

^1^Department of Gastric Surgery, Sun Yat-sen University Cancer Center; State Key Laboratory of Oncology in South China; Collaborative Innovation Center for Cancer Medicine, Guangzhou 510060, Guangdong, P. R. China;

^2^Department of Thoracic Surgery, Sun Yat-sen University Cancer Center; State Key Laboratory of Oncology in South China; Collaborative Innovation Center for Cancer Medicine, Guangzhou 510060, Guangdong, P. R. China;

^3^Department of Clinical Oncology, the University of Hong Kong, Hong Kong 999077, P. R. China;

^4^School of Biological Sciences, the University of Hong Kong, Hong Kong 999077, P. R. China;

^5^State Key Laboratory of Oncology in South China, Sun Yat-sen University Cancer Center; Collaborative Innovation Center for Cancer Medicine, Guangzhou 510060, Guangdong, P. R. China;

^6^Shenzhen Key Laboratory of Translational Medicine of Tumor and Cancer Research Centre, Shenzhen University, Shenzhen 518052, Guangdong, P. R. China;

^7^Department of Clinical Oncology, the First Affiliated Hospital, Zhengzhou University, Zhengzhou 450003, Henan, P. R. China.

^*^**Correspondence:** [zhangly1@sysucc.org.cn](mailto:zhangly1@sysucc.org.cn); [xyguan@hku.hk](mailto:xyguan@hku.hk).

**Table S1. Recombinant proteins and neutralizing antibodies used in this study.**

(Excel file)

**Table S2. Antibodies used in this study.**

(Excel file)

**Table S3. shRNAs used in this study.**

(Excel file)

**Table S4. Primers used in this study.**

(Excel file)

**Table S5. Gene Sets for GSEA analysis.**

(Excel file)

**Table S6. Motif enrichment analysis result of CAF-specific extracellular component encoded genes.**

(Excel file)

**Table S7. Summary of sequencing statistics and mapping of reads for 2 paired FGFR2^+^ fibrocyte and CAF sample pools.**

**(Excel file)**

**Table S8. Gene set of CAF-specific extracellular component used in motif enrichment analysis.**

(Excel file)

**
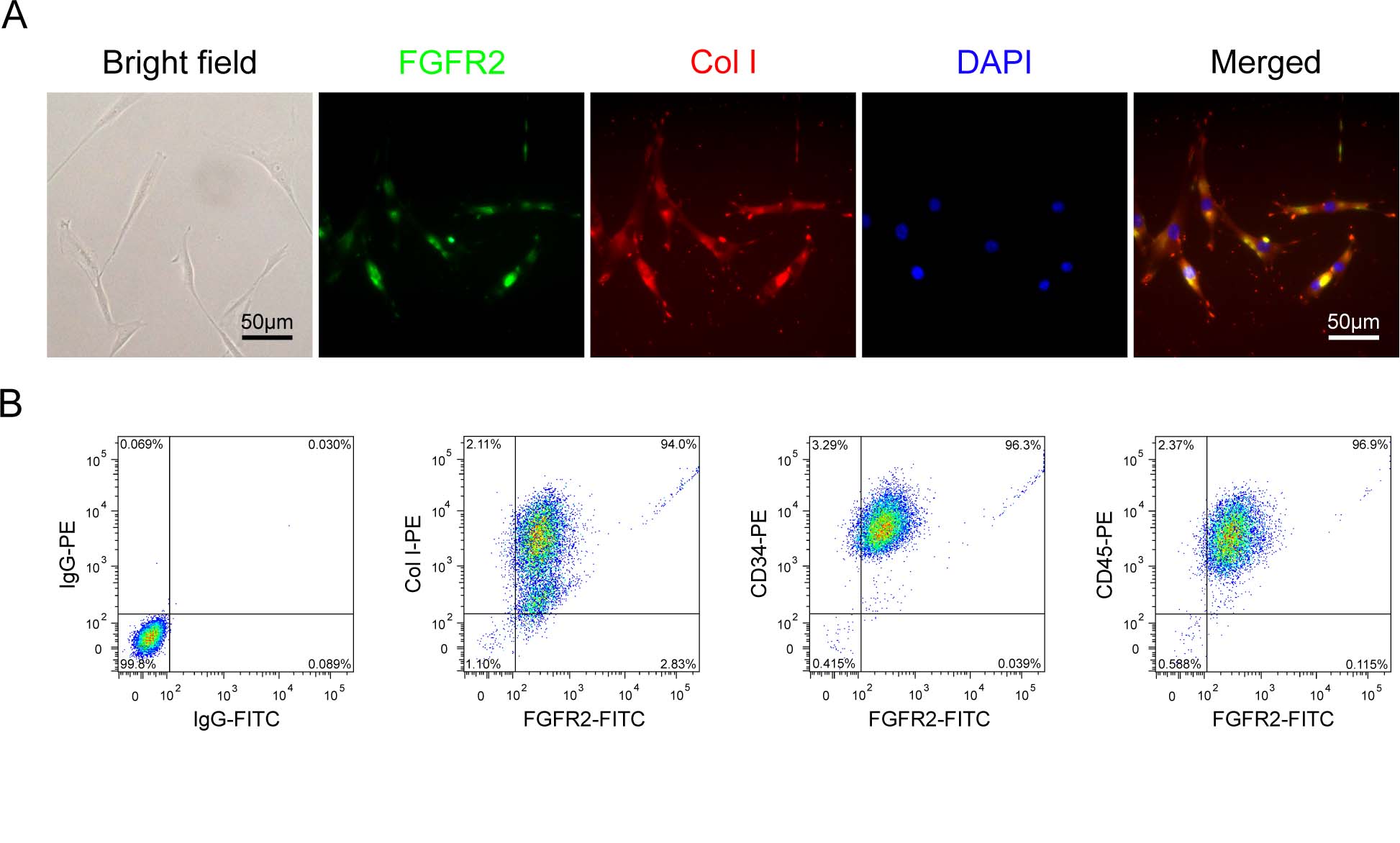
**

**Fig. S1 Characterization of fibrocyte isolates.**

**A.** Fibrocyte isolates were analyzed by IF staining using anti-FGFR2 (green) and anti-Collagen I (red) antibodies. Nuclei were labeled with DAPI (blue). Representative images of IF and light microscopic view (Bright field) are shown.

**B.** The purity of isolated fibrocytes was determined by FCM with FGFR2 and CD34/CD45/Collagen I double staining.

**Abbreviations:** IF, immunofluorescence; FCM, flow cytometry.


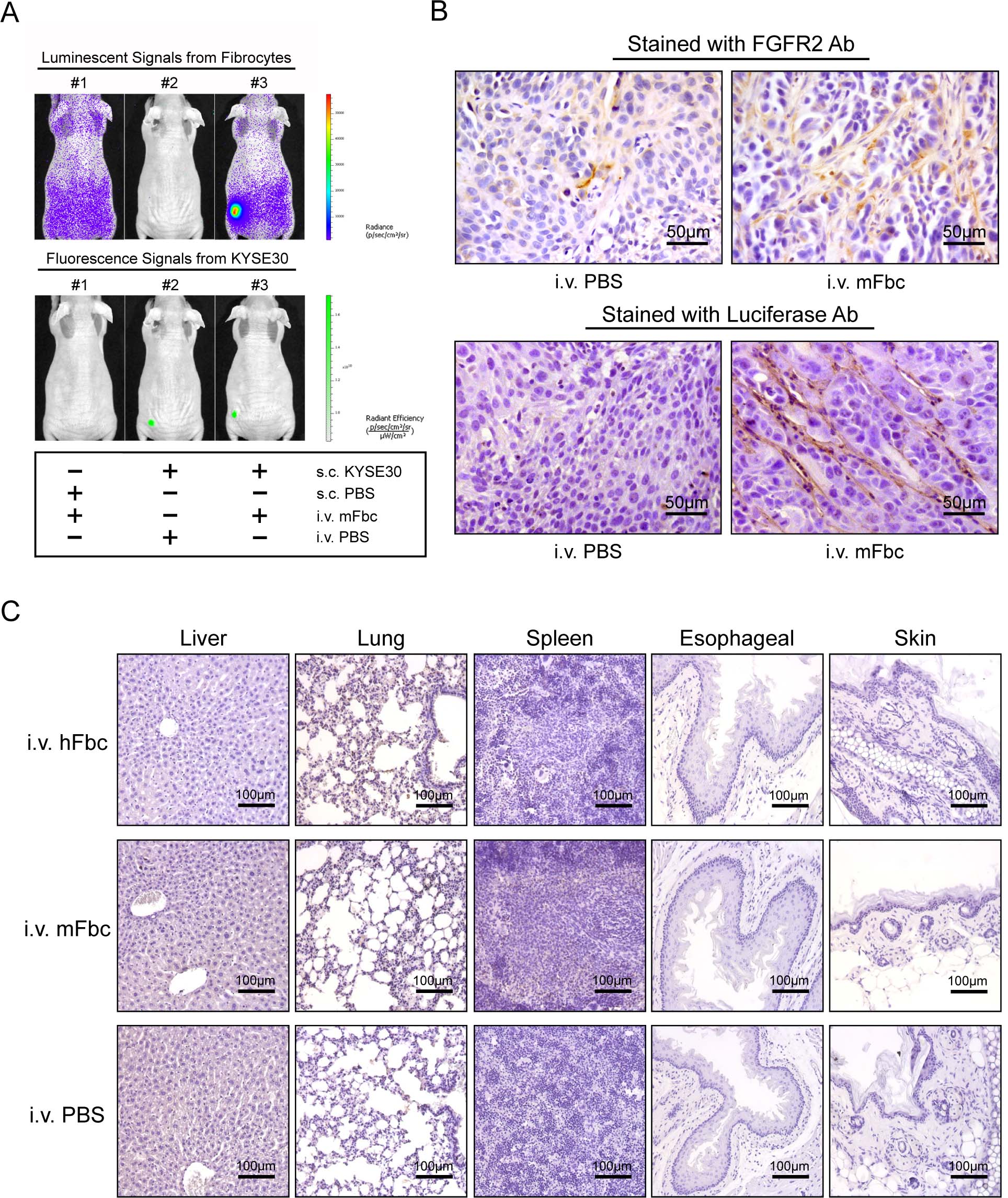


**Fig. S2 FGFR2^+^ circulating fibrocytes can be recruited into ESCC tumor mass.**

**A.** Luciferase-expressing murine fibrocytes (mFbcs) were subjected to the *in vivo* chemotaxis assays in tumor-bearing mice (#3; s.c. injection of GFP-expressing KYSE30) or control mice (#1; s.c. injected with PBS). mFbcs distribution (upper panel) and tumor burden (lower panel) were determined respectively at 24 hours after i.v. injection of mFbcs. Tumor-bearing mice with PBS i.v. injection (#2) were used to discard non-specific background signals. Three mice will be evaluated for each condition.

**B.** IHC staining demonstrates the presence of FGFR2^+^ cells or exogenous cells (Luciferase^+^ cells) in the xenograft tumors obtained from the nude mice in chemotaxis assays.

**C.** IHC staining demonstrates the presence of exogenous cells (Luciferase^+^ cells) in indicated organs obtained from the nude mice in chemotaxis assays.

**Abbreviations:** mFbc, murine fibrocyte; s.c., subcutaneous; i.v., intravenous; IHC, immunohistochemistry.


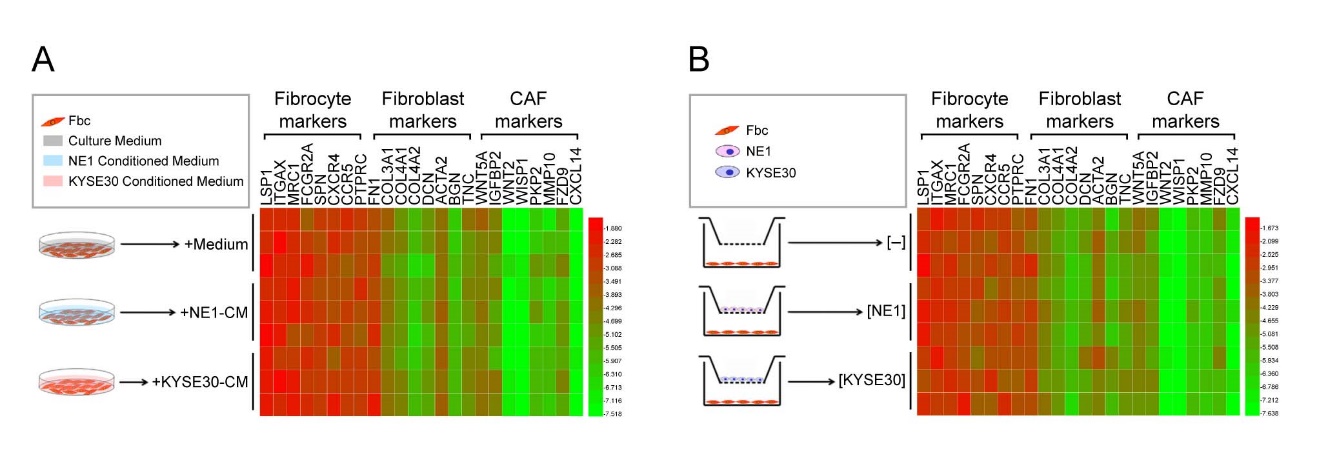


**Fig. S3 Paracrine factors produced by ESCC cells can not trigger the differentiation of fibrocytes.**

**A.** Expression of indicated marker genes in Fbcs treated with CM of KYSE30 (+KYSE30-CM) were determined by qPCR and summarized in heat map. The cells treated with normal culture medium (+Medium) or CM of NE1 (+NE1-CM) were used as controls. The experiment was repeated three times.

**B**. Expression of indicated marker genes in Fbcs co-cultured with KYSE30 ([KYSE30]) using transwell systems were determined by qPCR and summarized in heat map. The cells cultured with normal culture medium ([-]) or indirect co-cultured with NE1 ([NE1]) were used as controls. The experiment was repeated three times. Schematic diagrams in A-B showing the experimental procedures of differentiation assays. Color intensity of heat map represents log10 (Relative expression) within each measurement.

**Abbreviations:** Fbc, fibrocyte; CM, conditioned medium; qPCR, semi-quantitative PCR.


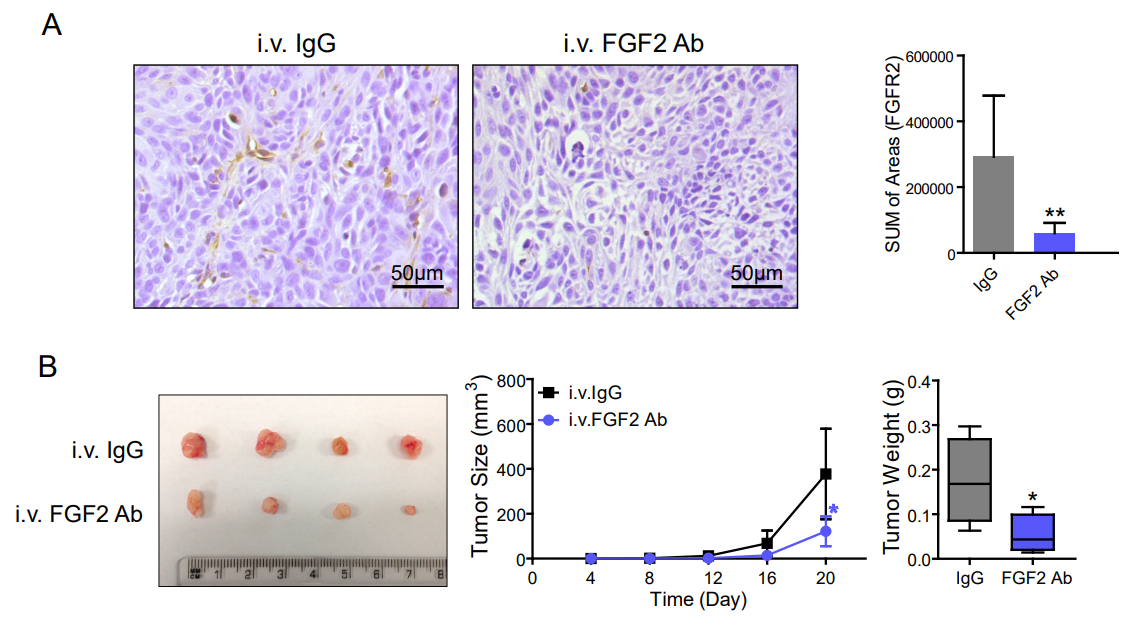


**Fig. S4 Treatment with FGF2 neutralizing antibody reduces tumor growth in mice model.**

**A.** The presence of FGFR2^+^ cells (left panel) in the EC9706 xenograft tumors obtained from the nude mice with FGF2 neutralizing antibody/control IgG treatment were detected by IHC staining and representative images are shown. The immunostaining area of FGFR2 was summarized in the right panel. The data are represented as mean ± SD in 4 mice. **, *P* <0.01.

**B.** The therapeutic effect of FGF2 neutralizing antibody was evaluated on EC9706 xenograft-bearing mice. Mice were treated intravenously everyday with FGF2 neutralizing antibody (4 μg/kg), or control IgG. Xenograft tumors were harvested at day 20 (left panel). Tumor volume and tumor weight was summarized in the middle panel and right panel. The data are represented as mean ± SD in 4 mice. *, *P* <0.05.

**Abbreviations:** IHC, immunohistochemistry.


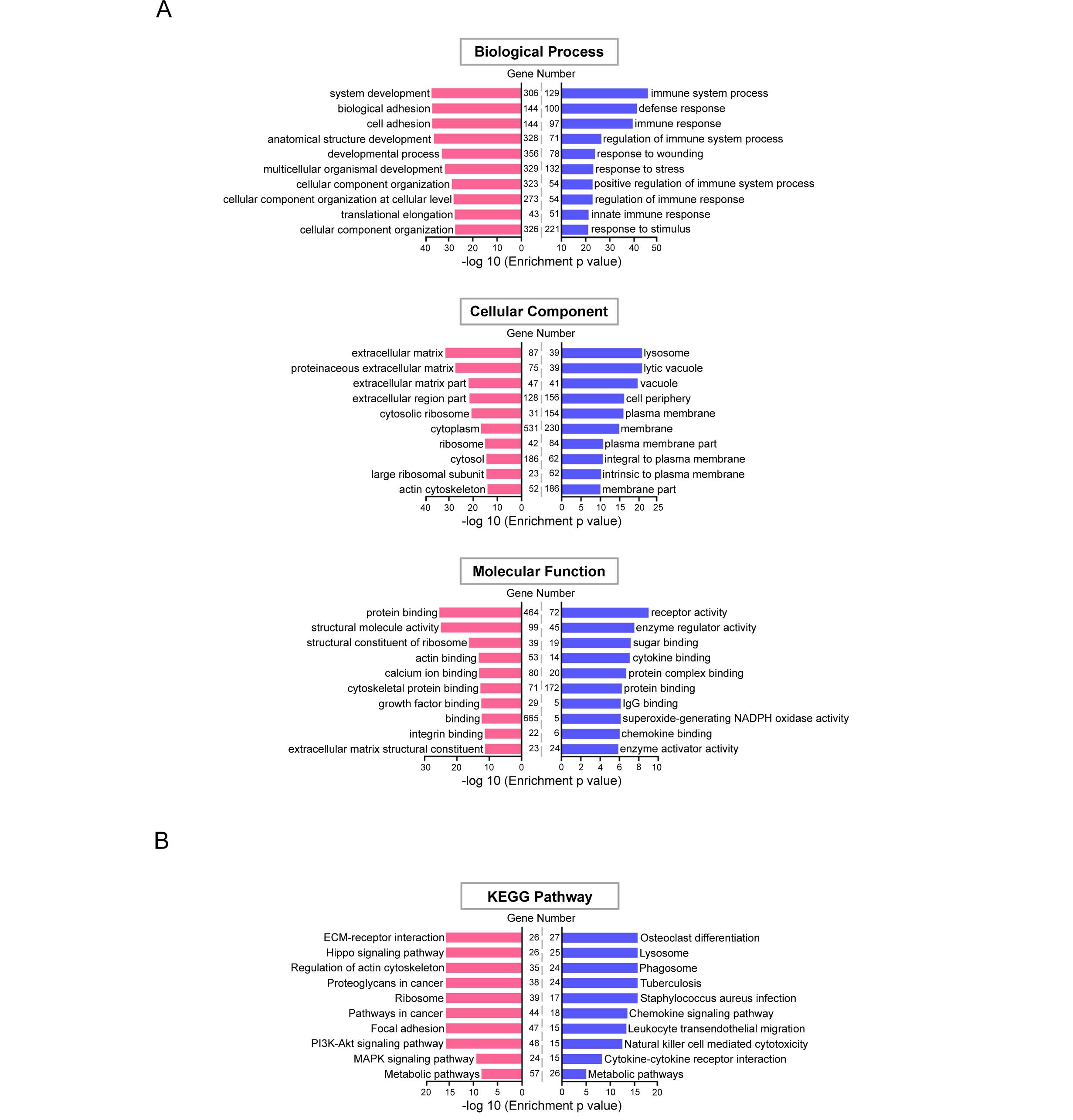


**Fig. S5 GO and KEGG pathway enrichment analysis of differentially expressed genes.**

**A**. Indicated are the top 10 enriched GO terms associated with biological process, cellular component, and molecular function for all upregulated (the enrichment *P*-value and gene number of each term were summarized in pink horizontal bar chart) and downregulated genes (the enrichment *P*-value and gene number of each term were summarized in blue horizontal bar chart).

**B**. Indicated are the top 10 enriched KEGG pathways for all upregulated (the enrichment *P*-value and gene number of each term were summarized in pink horizontal bar chart) and downregulated genes (the enrichment *P*-value and gene number of each term were summarized in blue horizontal bar chart).


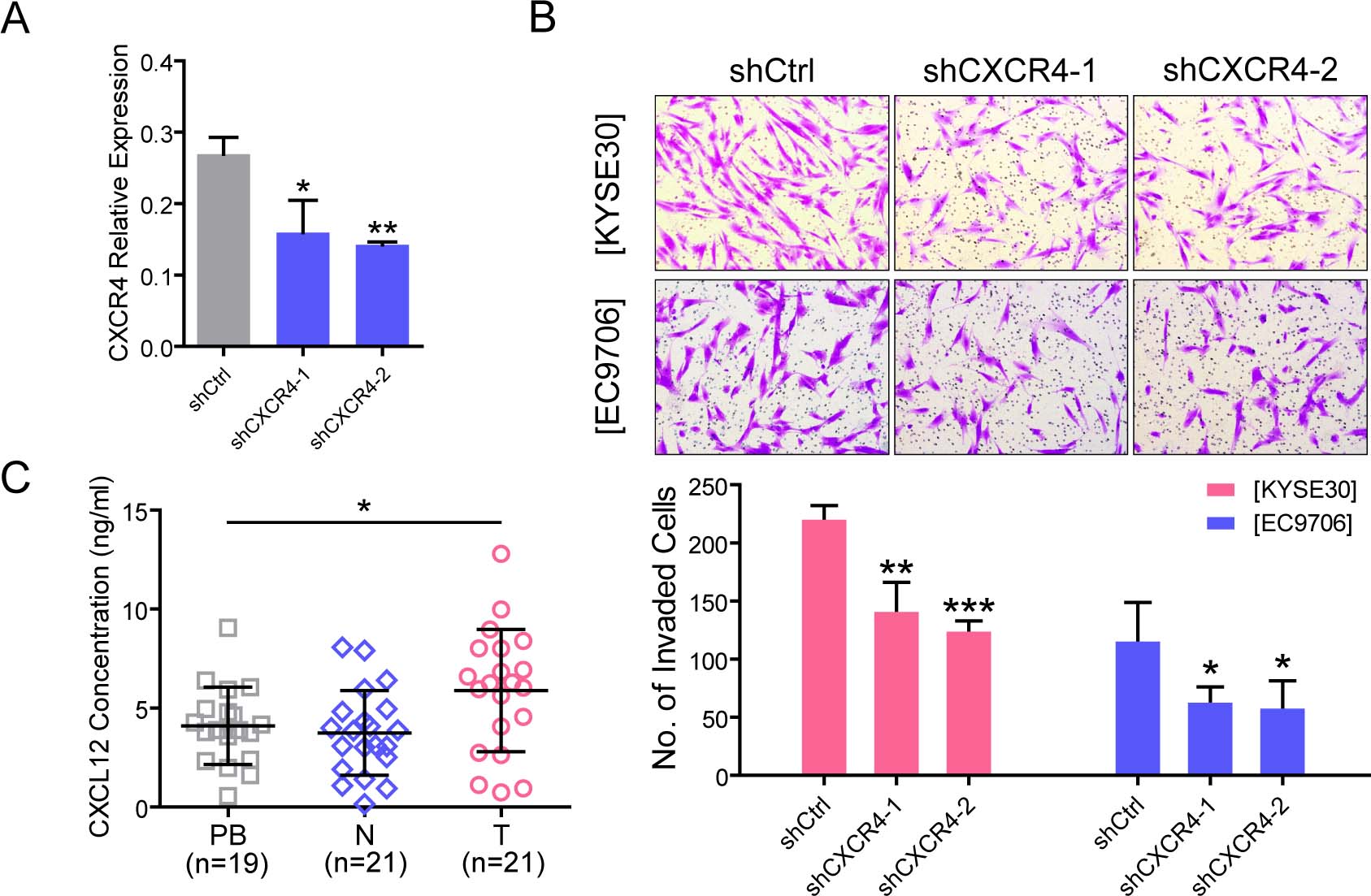


**Fig. S6 CXCL12/CXCR4 axis contributes to the recruitment of FGFR2^+^ fibrocytes in ESCC.**

**A**. CXCR4 relative expression in CXCR4 knockdown hFbcs (shCXCR4-1 and shCXCR4-2) or scramble-control hFbcs (shCtrl) were determined by qPCR. The data are represented as mean ± SD on 3 independent experiments. *, *P* <0.05; **, *P* <0.01.

**B.** CXCR4 knockdown hFbcs and control hFbcs were subjected to *in vitro* chemotaxis assays in the presence of indicated cell lines ([KYSE30] or [EC9706]) as chemotactic stimulus. Representative images of hFbcs invaded through matrigel are shown in the upper panel. Number of invaded hFbcs per 40× field was quantified in the lower panel. The data are represented as mean ± SD on 3 independent experiments. *, *P* <0.05; **, *P* <0.01; ***, *P* <0.001.

**C.** CXCL12 concentrations in plasma samples of ESCC patients (PB; *n* = 19), primary ESCC tissues (T; *n* = 21) and their corresponding non-tumor tissues (N; , *n* = 21) were detected by ELISA. Horizontal lines represent the median. The whiskers extend to the highest values excluding outliers and extremes. *, *P* <0.05.

**Abbreviations:** ESCC, esophageal squamous cell carcinoma; hFbc, human fibrocyte; Ctrl, control; qPCR, semi-quantitative PCR; ELISA, enzyme-linked immunosorbent assay.


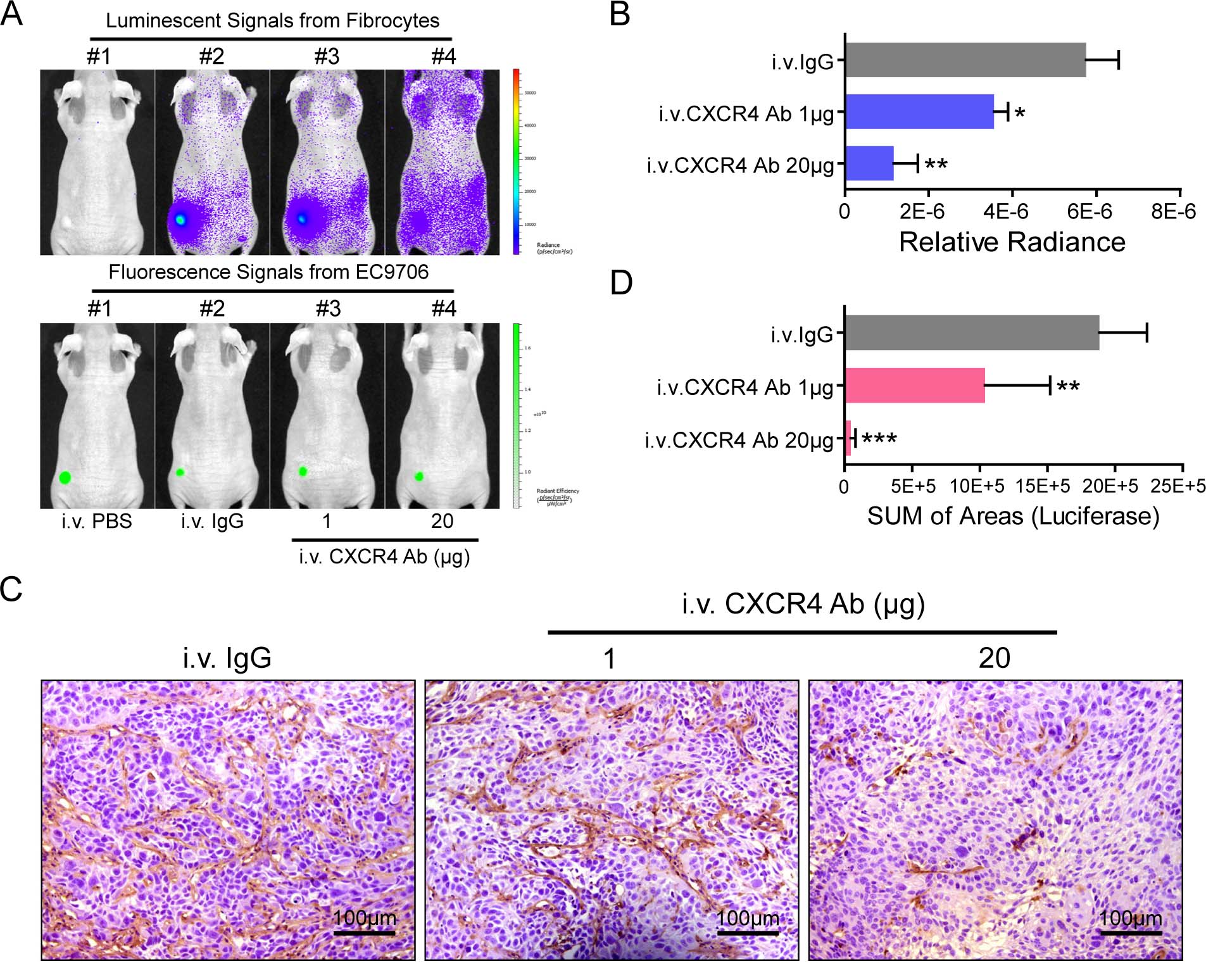


**Fig. S7 CXCR4 neutralizing antibody inhibit the recruitment of FGFR2^+^ fibrocytes to EC9706 xenograft tumors.**

**A-B**. The blocking effect of CXCR4 neutralizing antibody on the ESCC-induced chemotaxis was evaluated by *in vivo* chemotaxis assay. 1 hour after i.v. injection with Luciferase-expressing hFbcs, tumor bearing mice (generated by GFP-expressing EC9706) were treated with increasing doses of CXCR4 neutralizing antibody (#3 and #4) or control IgG (#2). hFbcs distribution (upper panel in A) and tumor burden (lower panel in A) were determined respectively at 24 hours after CXCR4 neutralizing antibody injection. Tumor-bearing mice treated with PBS (#1) were used to discard non-specific background signals. The relative radiance was summarized in histogram. The data are represented as mean ± SD on in 4 mice. *, *P* <0.05; **, *P* <0.01.

**C-D**. Representative IHC images of exogenous cells (Luciferase^+^ cells) in EC9706 xenografts obtained from the nude mice treated with CXCR4 neutralizing antibody or control IgG. The immunostaining area of Luciferase was summarized in D. The data are represented as mean ± SD in 4 mice. **, *P* <0.01; ***, *P* <0.001.

**Abbreviations:** ESCC, esophageal squamous cell carcinoma; i.v., intravenous; hFbc, human fibrocyte; IHC, immunohistochemistry.
